# *Drosophila S6 Kinase Like* Inhibits Neuromuscular Junction Growth by Downregulating the BMP Receptor Thickveins

**DOI:** 10.1371/journal.pgen.1004984

**Published:** 2015-03-06

**Authors:** Guoli Zhao, Yingga Wu, Li Du, Wenhua Li, Ying Xiong, Aiyu Yao, Qifu Wang, Yong Q. Zhang

**Affiliations:** 1 Key Laboratory of Molecular and Developmental Biology, Institute of Genetics and Developmental Biology, Beijing, China; 2 College of Life Science, Hubei University, Wuhan, China; Harvard Medical School & Children’s Hospital Boston, UNITED STATES

## Abstract

Synaptic connections must be precisely controlled to ensure proper neural circuit formation. In *Drosophila melanogaster*, bone morphogenetic protein (BMP) promotes growth of the neuromuscular junction (NMJ) by binding and activating the BMP ligand receptors wishful thinking (Wit) and thickveins (Tkv) expressed in motor neurons. We report here that an evolutionally conserved, previously uncharacterized member of the S6 kinase (S6K) family S6K like (S6KL) acts as a negative regulator of BMP signaling. *S6KL* null mutants were viable and fertile but exhibited more satellite boutons, fewer and larger synaptic vesicles, larger spontaneous miniature excitatory junctional potential (mEJP) amplitudes, and reduced synaptic endocytosis at the NMJ terminals. Reducing the gene dose by half of *tkv* in *S6KL* mutant background reversed the NMJ overgrowth phenotype. The NMJ phenotypes of *S6KL* mutants were accompanied by an elevated level of Tkv protein and phosphorylated Mad, an effector of the BMP signaling pathway, in the nervous system. In addition, Tkv physically interacted with S6KL in cultured S2 cells. Furthermore, knockdown of S6KL enhanced Tkv expression, while S6KL overexpression downregulated Tkv in cultured S2 cells. This latter effect was blocked by the proteasome inhibitor MG132. Our results together demonstrate for the first time that S6KL regulates synaptic development and function by facilitating proteasomal degradation of the BMP receptor Tkv.

## Author Summary

Bi-directional signaling between neurons and their target cells is critical for synapse formation, growth, and plasticity, as well as for neuronal survival. Bone morphogenetic protein (BMP) acts as a retrograde signal promoting synaptic growth at the *Drosophila* neuromuscular junction (NMJ), but little is known about proteins that regulate BMP signaling by controlling BMP release, receptor expression, and signal transduction. We report here that a previously uncharacterized and evolutionally conserved member of the S6 kinase (S6K) family S6K like (S6KL) inhibits BMP signaling by interacting with and promoting proteasome-mediated degradation of the BMP receptor Thickveins (Tkv). In *S6KL* mutants, there was an elevated level of Tkv protein, together with overgrown NMJs characterized by excess satellite boutons. Reducing the gene dose of *tkv* by half in *S6KL* null background restored normal NMJ morphology, suggesting that S6KL normally serves to suppress Tkv-mediated BMP signaling. Biochemically, S6KL interacted with Tkv. Overexpression of S6KL down-regulated Tkv and this effect was inhibited by blocking the proteasomal degradation pathway. Collectively, our data demonstrate that S6KL regulates NMJ synapse development by promoting the proteasomal degradation of Tkv. Thus, we have identified a novel negative regulator of BMP signaling in the *Drosophila* nervous system.

## Introduction

Reliable and effective communication between neurons and their targets across the synaptic cleft through cell adhesion molecules and signaling pathways is critical for the formation, growth, and plasticity of synapses [1–3]. For example, the retrograde bone morphogenetic protein (BMP) signaling from postsynaptic muscles to presynaptic motoneurons is crucial for synaptic development and plasticity at the *Drosophila* larval neuromuscular junctions (NMJs) [4–8]. A similar role of BMP signaling has also been identified at the central synapses in vertebrates [9].

At the *Drosophila* NMJ, the BMP homolog Glass bottom boat (Gbb) is secreted from muscles and binds to the constitutively active presynaptic BMP type II receptor wishful thinking (Wit), which in turn leads to the recruitment of the type I receptors thickveins (Tkv) and saxophone (Sax). Wit phosphorylates and activates Tkv and Sax, which in turn phosphorylate mothers against decapentaplegic (Mad). Phosphorylated Mad (pMad) forms a complex with co-Smad Medea that translocates to the nucleus and regulates the transcription of target genes required for NMJ growth. Mutation of any member of this cascade leads to a drastic reduction in the number of synaptic boutons and the amount of neurotransmitter released at the NMJ [10–13]. Conversely, up-regulation of BMP signaling leads to NMJ overgrowth [8,14–17]. Thus BMP pathway is both required and sufficient for *Drosophila* NMJ growth.

At the *Drosophila* NMJ, BMP signaling is tightly regulated at multiple levels. For example, BMP signaling is attenuated by endocytosis and endosomal trafficking of BMP receptors Wit and Tkv [14–19]. Recently, we uncovered that brain tumor (Brat), a translational suppressor, suppresses Mad expression in motoneurons [8]. We report here that an evolutionally conserved and previously uncharacterized kinase, namely a protein kinase encoded by a gene mapped at chromosome band 17E (PK17E), acts as a negative regulator of Tkv in regulating NMJ synapse growth. Based on sequence homology and kinase activity assays, we renamed PK17E as S6KL, short for ribosomal protein S6 kinase like.

We report here that *S6KL* null mutation causes NMJ overgrowth characterized by excess satellite boutons. This NMJ overgrowth is caused by up-regulation of BMP signaling based on genetic and immunochemical analyses. We further show that S6KL physically interacts with Tkv and promotes its proteasomal degradation, resulting in downregulation of BMP signaling and inhibition of NMJ growth. Thus, our study identifies a novel regulatory mechanism of BMP signaling in the *Drosophila* nervous system.

## Results

### Isolation and characterization of *S6KL* mutants

**Fig 1:**
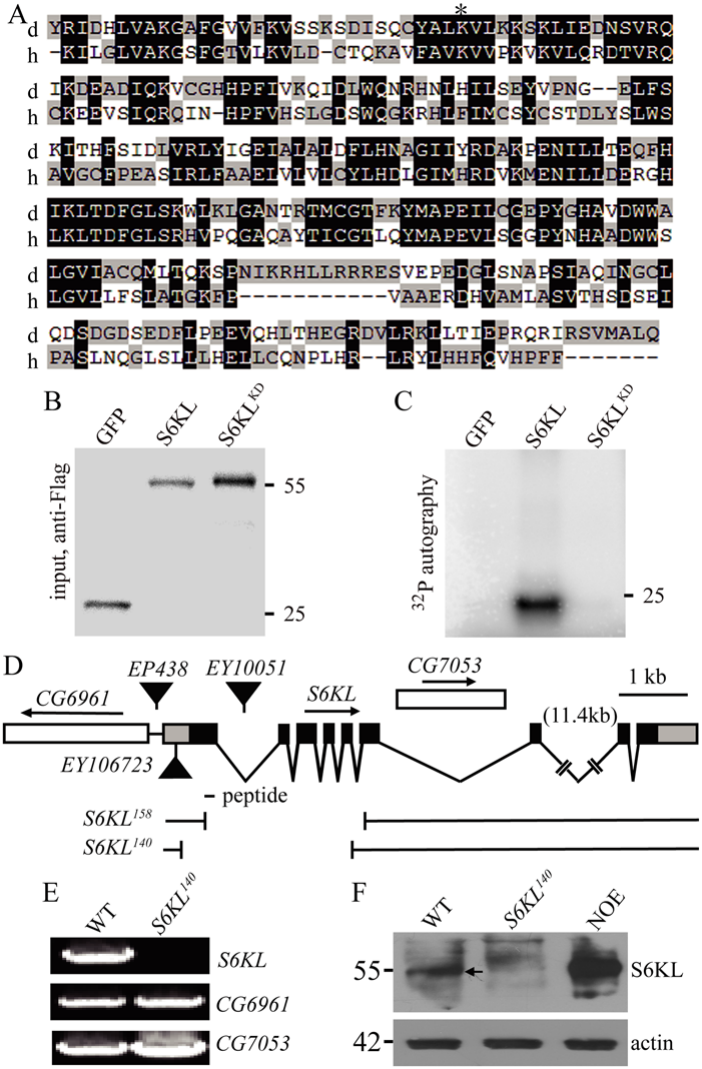
Kinase domain alignment of S6KL and its human homolog and molecular characterization of *S6KL* mutants. (A) Kinase domain sequence alignment of *Drosophila* S6KL (d) and human SGK494 (h). Dark and light gray shading indicate identical and similar amino acids, respectively. The conserved lysine for ATP binding is indicated by an asterisk. (B, C) The kinase activity assay for S6KL. S2 cells were transfected with expression vectors for Flag-GFP, Flag-S6KL, or Flag-S6KL^K193Q^. Cell lysates were immunoprecipitated by anti-Flag antibody. The immunoprecipitates were divided into two portions, one for western analysis of inputs detected with anti-Flag (B), the other for in vitro kinase assay myelin basic protein (MBP) as substrates (C). The protein band in C indicates phosphorylated MBP. Numbers on the right are molecular masses in kilo-Daltons. (D) Genomic structure of *S6KL* and mapping of mutants. The intron-exon organization of *S6KL* and its flanking *CG6961* and nesting *CG7053* are shown, so is the peptide for anti-S6KL antibody production. Black boxes, coding regions; gray boxes, untranslated regulatory regions; gaps, introns. The deletion lines *S6KL* ^*158*^ and *S6KL* ^*140*^ generated by imprecise excision of *EY10051* are depicted. (E) RT-PCR analysis of *S6KL*, *CG6961*, and *CG7053* from whole adult flies of wild type (WT) and *S6KL* ^*140*^ mutants. (F) Western blots of adult head protein extracts from wild type, *S6KL* ^*140*^, and neuronal overexpression of S6KL in wild-type background (NOE; *elav-Gal4/+; UAS-S6KL/+*) probed with anti-S6KL. The arrow indicates S6KL protein from wild type head extracts. Actin was used as a loading control.

To better understand the neuronal functions of *Drosophila* fragile X mental retardation protein (dFMRP) in *Drosophila*, we screened a *Drosophila* embryo cDNA library with the yeast two-hybrid system using the N-terminal 220 amino acids peptide of dFMRP as a bait. We identified three positive genes including *PK17E* represented by one clone encoding the 98 amino acid residues of PK17E C-terminal followed by 272 bp 3’-UTR sequence. Since dFMRP regulates NMJ development, we examined the synaptic role of *PK17E* for which there were mutants surviving to the third instar larvae. Though the candidate clone for *S6KL* turned out to be a false positive as the clone alone without bait expression was sufficient to activate the reporter expression, we found that multiple *S6KL* mutants, including trans-allelic *EY06723* and *EY10051* with a *P* element inserted in the genomic *S6KL* gene (Fig. 1D) or hemizygotes of each insertion, showed obvious NMJ overgrowth phenotype. On the contrary, overexpression of S6KL was reported exhibiting fewer synaptic boutons from a gain-of-function screen for genes controlling motor axon guidance and synaptogenesis in *Drosophila* [20]. Thus, we suspected that S6KL played a role in synapse development and characterized its synaptic function in the present study.

Sequence comparison showed that PK17E is a member of the S6K family and contains a single serine/threonine protein kinase (STK) domain with 36% identity and 52% similarity, and 33% identity and 48% similarity to the STK domain of human S6K1 and *Drosophila* dS6K, respectively. Given that the previously characterized *Drosophila* S6K (dS6K) is highly homologous to human S6K1 and S6K2 within the STK domain (83% identity), we renamed PK17E “S6K like” (S6KL). S6KL is well conserved from *C*. *elegans* to humans. Specifically, the STK domain of the *Drosophila* S6KL is 45% identical and 63% similar to that of the uncharacterized human homolog SGK494 (http://www.kinase.com/cgi-bin) (Fig. 1A). So far, the physiological functions of S6KL and its homologs have not been characterized in any organisms.

Sequence analysis revealed that S6KL contains a catalytic domain shared by the serine/threonine protein kinase A, G, and C (AGC) families categorized based on their tendency to phosphorylate sites surrounded by basic amino acids. To verify if S6KL has an intrinsic kinase activity, S6KL was tagged with a Flag epitope and expressed in S2 cells using an actin promoter. To control for nonspecific kinase activity, a putative kinase-dead mutant of S6KL, Flag-S6KL^K193Q^, in which lysine 193 was mutated to glutamine disrupting ATP binding and hence the kinase activity based on previous studies on S6 kinases [21,22] (Fig. 1A), and a vector control Flag-GFP were similarly expressed in S2 cells. Following transfection, S2 cells were lysed, and the cell lysates were subjected to immunoprecipitation with anti-Flag antibody (Fig. 1B). The immunoprecipitates were then assayed for kinase activity by detecting γ-^32^P incorporation from [γ-^32^P] ATP into a generic kinase substrate myelin basic protein (MBP). While GFP control and S6KL^K193Q^ showed no kinase activity, wild-type S6KL effectively phosphorylated MBP (Fig. 1C), demonstrating an intrinsic kinase activity for S6KL.

**Fig 2:**
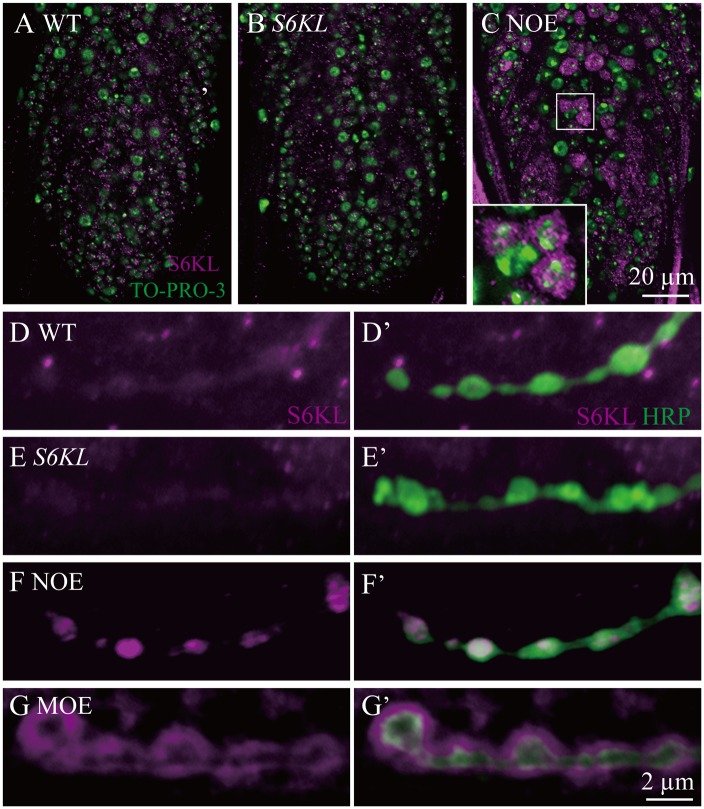
Overexpressed S6KL enriches in pre- and postsynaptic area. A–C, Representative staining results of a ventral nerve cord from wild type (A), *S6KL* ^*140*^ (B), and *OK6-Gal4/+; UAS-S6KL/+* (NOE; C) double-stained with anti-S6KL (magenta) and TO-PRO-3 (labeling nuclei; green). Scale bar, 20 μm. D–G’, Representative staining results of NMJ terminals double-stained with anti-S6KL and anti-HRP from wild type (D), *S6KL* ^*140*^ (E), *OK6-Gal4/+; UAS-S6KL/+* (NOE; F), and *C57-Gal4/UAS-S6KL* (MOE; G). Scale bar, 2 μm.

To investigate the in vivo functions of S6KL, we generated multiple *S6KL* mutant alleles by imprecise excision of *EY10051*. The *S6KL* ^*158*^ allele had a deletion of 2376 base pairs (bp), whereas *S6KL* ^*140*^ had a deletion of 2553 bp spanning the first to the fifth exon (Fig. 1D). Full-length *S6KL* mRNA was not detected in homozygous *S6KL* ^*140*^ adults (Fig. 1E), whereas mRNAs of the flanking *CG6961* and the intragenic *CG7053* were expressed normally (Fig. 1E). Western analysis with a rabbit polyclonal antibody against a peptide spanning amino acid residues 66–82 of S6KL (Fig. 1D) detected no target protein expression in homozygous *S6KL* ^*140*^ adult heads (Fig. 1F). *S6KL* ^*140*^ appeared to be a null allele, as it had an intragenic deletion removing the N-terminal 261 amino acid residues including part of the kinase domain and behaved as a null phenotypically (see below). Immunostaining showed no apparent expression of the endogenous S6KL in the ventral nerve cord (Fig. 2A and 2B), wing disc, and eye disc (S1 Fig.), but appreciable expression in a small subset of cells in the leg disc (S1 Fig.). When overexpressed driven by the motoneuron specific *OK6-Gal4*, S6KL was present primarily in the cytoplasm (Fig. 2C). Endogenous S6KL was not detectable at the NMJs, but overexpressed S6KL can localize to both presynapse and postsynapse when *UAS-S6KL* is under the control of the motor neuron specific *OK6-Gal4* and the muscular *C57-Gal4*, respectively (Fig. 2D–2G’). Homozygous *S6KL* ^*140*^ and *S6KL* ^*158*^ mutants were fully viable, fertile in both males and females, and showed no obvious developmental and behavioral abnormalities. In this study, we focused on characterizing the synaptic phenotypes of *S6KL* ^*140*^ mutants at NMJ terminals.

### Overgrown NMJ terminals in *S6KL* mutants

**Fig 3:**
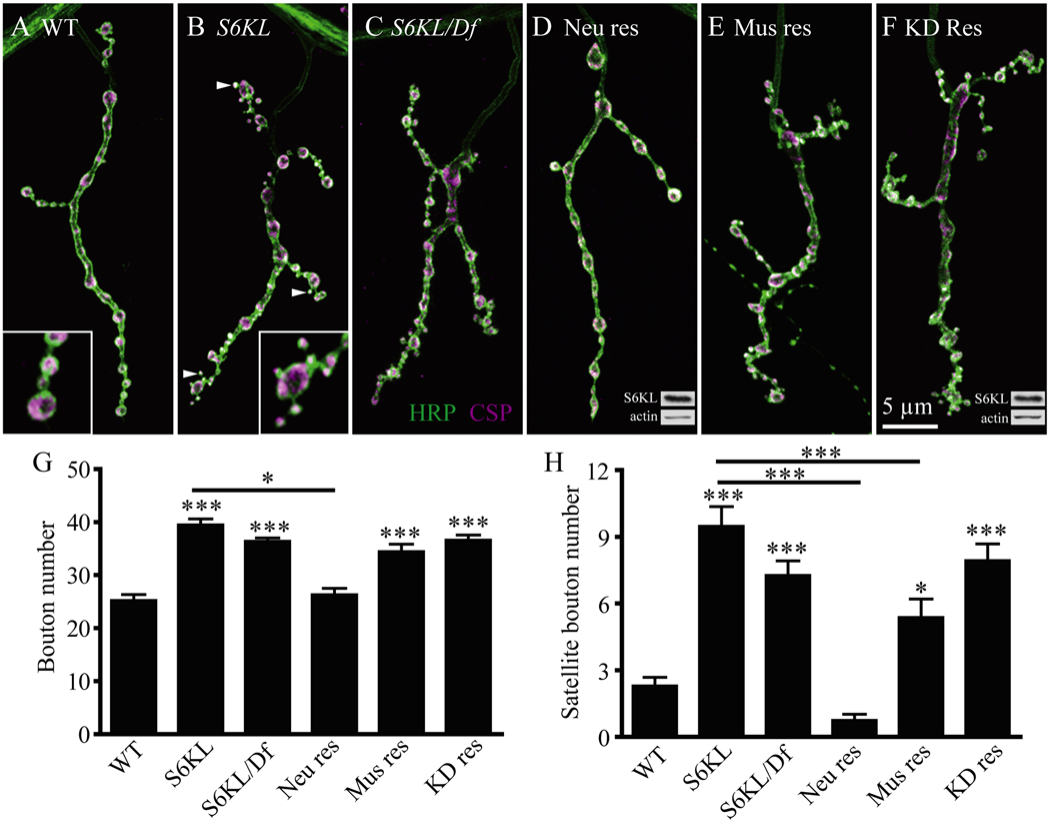
Overgrown NMJs in *S6KL* mutants. (A–F) Representative NMJ 4 synapses from different genotypes double-stained with anti-HRP recognizing neuronal plasma membrane (green) and an antibody against CSP (magenta), a synaptic vesicle protein. Insets show higher-magnification images of terminal boutons. The genotypes are: (A) Wild type, (B) homozygous *S6KL* ^*140*^ mutants, (C) hemizygous *S6KL* ^*140*^ */Df(1)ED741* mutants, (D) neuronal rescue of *S6KL* ^*140*^ by overexpression of S6KL under the control of *elav-Gal4* (*S6KL* ^*140*^ *elav-Gal4/S6KL* ^*140*^ *; UAS-S6KL/+*), (E) Muscular rescue of *S6KL* ^*140*^ by overexpression of S6KL under the control of *Mhc-Gal4* (*S6KL* ^*140*^; *UAS-S6KL/Mhc-Gal4*), and (F) Neuronal rescue of *S6KL* ^*140*^ by overexpression of the kinase dead S6KL (S6KL^K193Q^) under the control of *elav-Gal4* (*S6KL* ^*140*^ *elav-Gal4/ S6KL* ^*140*^ *; UAS-S6KL* ^*K193Q*^ */+*). Satellite boutons are indicated by arrows in B. Insets D and F show Western results of larvae brain extracts using anti-S6KL and anti-actin antibodies. Scale bar, 5 μm. (G, H) Statistical results of the number of total boutons (G) and satellite boutons (H) in different genotypes. *n* = 17, 19, 15, 16, 19 and 18 NMJs for wild type, *S6KL* ^*140*^, *S6KL* ^*140*^ */Df(1)ED7413*, *S6KL* ^*140*^ *elav-Gal4/S6KL* ^*140*^ *; UAS-S6KL/+*, *S6KL* ^*140*^; *UAS-S6KL/Mhc-Gal4*, and *S6KL* ^*140*^ *elav-Gal4/S6KL* ^*140*^ *; UAS-S6KL* ^*K193Q*^ */+*, respectively. ***p<0.001 by one-way ANOVA with Tukey post hoc test; error bars indicate SEM.

The *Drosophila* NMJ is an effective model system for studying synaptic development and function. To elucidate a possible role for *S6KL* at synapses, we first examined the NMJ morphology of *S6KL* mutants. The wild type muscle 4 NMJ (NMJ4) showed the stereotypical “beads-on-a-chain” morphology when co-stained with an antibody against cysteine string protein (CSP), a synaptic vesicle-associated protein, and anti-horse radish peroxidase (HRP) which labels neuronal membrane. The NMJ synapses in *S6KL* ^*140*^ mutants were obviously overgrown with extra boutons (Fig. 3A and 3B). The mean total number of synaptic boutons per NMJ4 was 39.47±1.14 in *S6KL* ^*140*^ mutants, significantly higher than 25.24±1.10 in wild types (*p*<0.001; Fig. 3A, 3B, and 3G). Satellite boutons are ectopic boutons that emerge from the main nerve terminal or bud from primary boutons [14,23]. Compared with wild type, the mean satellite bouton number was more than four-fold higher in *S6KL* ^*140*^ mutants (*p*<0.001; Fig. 3A, 3B, and 3H). The NMJ phenotype of hemizygous *S6KL* ^*140*^ */Df(1)ED7413* mutants (*Df(1)ED7413* deletes the *S6KL* locus completely) was similar to that of homozygous *S6KL* ^*140*^ mutants. Presynaptic expression of S6KL driven by the motoneuron-specific *elav-Gal4* in *S6KL* ^*140*^ background restored the overgrown NMJ to wild-type level (Fig. 3D, 3G and 3H), whereas *Mhc-Gal4*-driven postsynaptic expression of S6KL did not (Fig. 3E, 3G and 3H), suggesting a specific role in presynaptic neurons.

To investigate whether the kinase activity of S6KL is required for NMJ growth regulation, we carried out rescue experiments by expressing wild-type and the kinase-dead K193Q mutant S6KL in *S6KL* ^*140*^ background. The overgrown NMJs in *S6KL* mutants were fully rescued by presynaptic expression of wild-type but not S6KL^K193Q^ under the control of *elav-Gal4* (Fig. 3F–3H), though S6KL^K193Q^ was expressed at a substantial level as the wild-type S6KL (insets of Fig. 3D and 3F). These results demonstrate that the kinase activity of S6KL is required for inhibiting NMJ growth.

### S6KL restrains NMJ growth presynaptically

**Fig 4:**
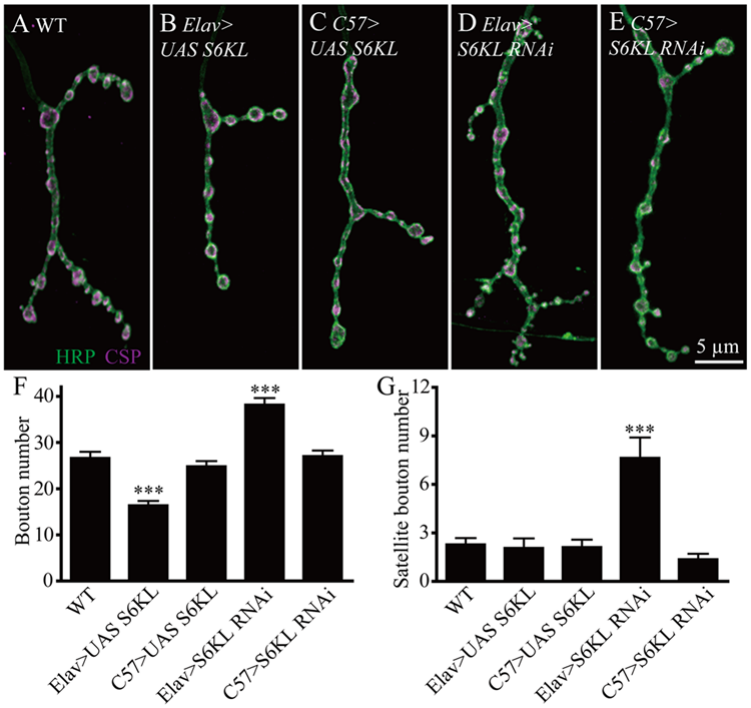
S6KL functions presynaptically in regulating NMJ growth. (A–E) Representative NMJ4 synapses from different genotypes doubly stained with anti-HRP (green) and anti-CSP (magenta). (A) wild type, (B) *elav-Gal4/+; UAS-S6KL/+*, (C) *C57-Gal4/UAS-S6KL*, (D) *elav-Gal4/+; S6KL RNAi/+*, and (E) *S6KL RNAi/+; C57-Gal4/+*. Scale bar, 5 μm. (F, G) Statistical results of the number of boutons (F) and satellite boutons (G) in different genotypes. *n* >16 for each genotype; ***p < 0.001 by one-way ANOVA with Tukey post hoc test; error bars indicate SEM.

The above-mentioned tissue-specific rescue results suggest a presynaptic function of S6KL. To further verify this notion, we carried out tissue-specific alterations of S6KL expression and examined their effects on NMJ growth. Presynaptic overexpression of S6KL by the pan-neuronal *elav-Gal4* resulted in a significant decrease in the number of total boutons (26.67±1.32 boutons for WT and 16.43±0.95 boutons for *elav-Gal4 > UAS-S6KL*; *p*<0.001; Fig. 4A, 4B and 4F), consistent with a previous report [20]. Whereas overexpression of S6KL in postsynaptic muscles using *C57-Gal4* had no effect on bouton number (26.67±1.32 boutons for WT and 24.86±1.13 boutons for *C57-Gal4* > *UAS-S6KL*; Fig. 4A, 4C, and 4F). Targeted knockdown of S6KL in presynaptic neurons by *elav-Gal4*-driven RNAi (VDRC line 102179) resulted in a significant increase in the number of total and satellite boutons compared with wild type (26.67±1.32 boutons for WT and 38.22±1.41 boutons for *elav-Gal4* > *RNAi*; *p*<0.001; Fig. 4A, 4D, 4F and 4G), whereas RNAi knockdown of S6KL in postsynaptic muscles by *C57-Gal4* showed no effect on bouton number (26.67±1.32 boutons for WT and 27.06±1.19 boutons for *C57-Gal4* > *RNAi*; Fig. 4A, 4E–4G). Similar results were observed using a second VDRC RNAi line 47326 targeting an independent sequence (26.67±1.32 boutons for WT and 41.04±1.33 boutons for *elav-Gal4* > *RNAi 47326*; *p*<0.001). These findings demonstrate that S6KL inhibits NMJ growth presynaptically.

### Fewer but larger synaptic vesicles in *S6KL* mutant boutons

**Fig 5:**
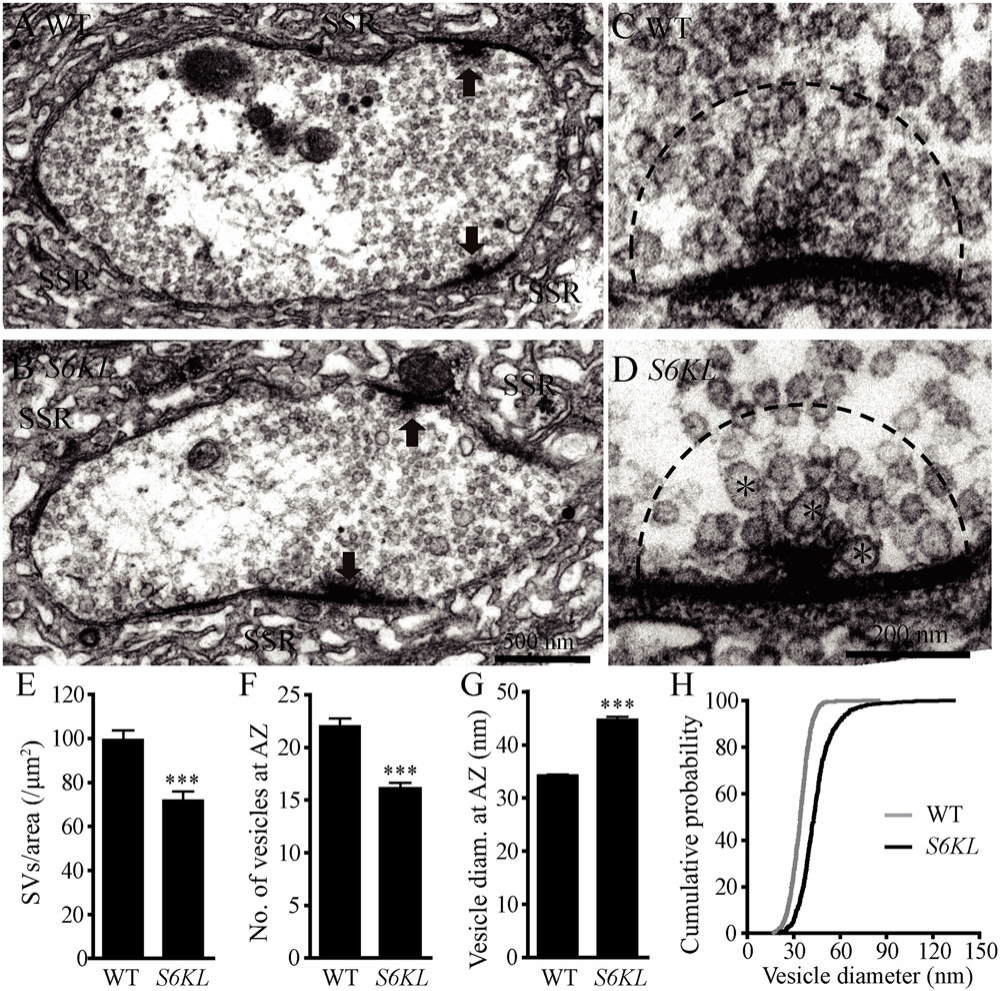
Fewer but larger synaptic vesicles at the active zone of *S6KL* mutant boutons. (A–D) Ultrastructure of wild type (A and C) and *S6KL* ^*140*^ mutant (B and D) NMJ boutons. (A and B) Transmission electron micrographs of synaptic boutons. Arrows indicate active zones with T bars; SSR, sub-synaptic reticulum. Scale bar, 500 nm. (C and D) Higher-magnification images of active zones. Fewer and larger synaptic vesicles are present in *S6KL* ^*140*^ mutant active zones compared with wild type. Large synaptic vesicles are indicated by asterisks. Scale bar, 200 nm. (E–G) Quantification of the ultrastructural features: synaptic vesicle density in the whole presynaptic bouton area (E), the number (F) and diameter (G) of synaptic vesicles in a 200 nm radius around the T-bar. For the analysis of SV density, *n* = 73 and 46 boutons for wild type and *S6KL* ^*140*^, respectively. For the analysis of SV in a 200 nm radius around T-bar, *n* = 40 and 34 boutons for wild type and *S6KL* ^*140*^, respectively. ***p<0.001 by Student’s *t*-test; error bars indicate SEM. (H) Cumulative histograms of synaptic vesicle diameter indicating larger vesicles in *S6KL* ^*140*^ mutants.

To investigate further the effect of S6KL on the ultrastructure of NMJs, we examined NMJ morphology by transmission electron microscopy. Presynaptic structures essential for neurotransmitter release at NMJ terminals include synaptic vesicles (SVs) and active zones with T bars (arrows in Fig. 5A and 5B), while the most prominent postsynaptic structure is the sub-synaptic reticulum (SSR) composed of a meshwork of convoluted muscle plasma membrane (Fig. 5A and 5B). The postsynaptic SSR appeared largely normal in *S6KL* ^*140*^ mutants (compare Fig. 5B with 5A). However, the SV density within the whole presynaptic bouton was 71.85±3.96 SVs/μm^2^ in *S6KL* ^*140*^ mutants, moderately but significantly reduced than 99.39±4.14 SVs/μm^2^ in wild type (*p*<0.001; Fig. 5A, 5B, and 5E). A small subpopulation of vesicles were larger in *S6KL* ^*140*^ mutants (asterisks in Fig. 5D). Statistical analysis of the density and size of SVs within a 200 nm radius around active zones showed that there were 16.09±0.57 SVs in *S6KL* ^*140*^ mutants, fewer than 22.03±0.74 SVs in wild type (*p*<0.001; Fig. 5C, 5D, and 5F), whereas the mean SV diameter was 44.75±0.53 nm in *S6KL* ^*140*^ mutants, significantly larger than 34.23±0.22 nm in wild type (*p*<0.001; Fig. 5G). A cumulative probability plot showed that 80% of wild-type SVs were < 40 nm, compared with only 35% of SVs < 40 nm in diameter in *S6KL* ^*140*^ mutants (Fig. 5H). Hence, loss of *S6KL* led to reduced vesicle density, and fewer and larger SVs at the neurotransmitter release sites in NMJ terminals, phenotypes similar to that observed in many endocytic mutants such as *brat*, *endophilin*, *synaptojanin*, and *AP180/lap* [8,24–26].

### Normal evoked neurotransmission but increased quantal size in *S6KL* mutants

**Fig 6:**
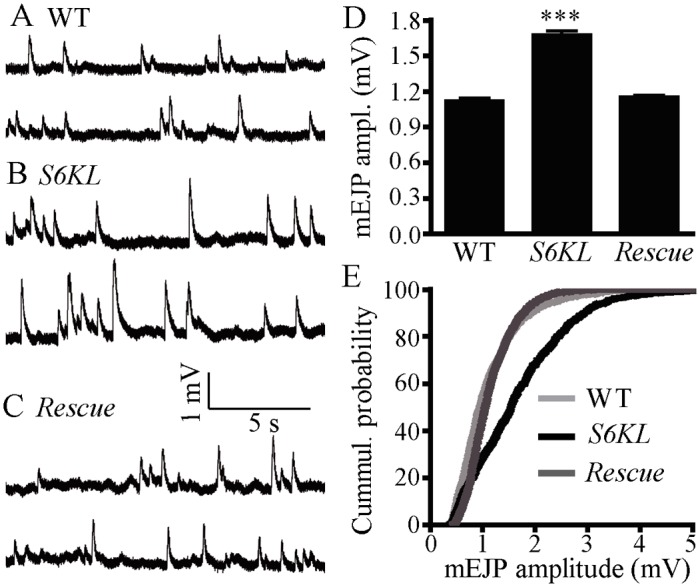
Quantal size is increased in *S6KL* mutants. (A–C) Representative recordings of mEJP from wild type (A), *S6KL* ^*140*^ (B), and neuronal rescue of *S6KL* ^*140*^ mutants (*S6KL* ^*140*^ *elav-Gal4/ S6KL* ^*140*^ *; UAS-S6KL/+*) (C). Muscle cell mEJPs were recorded in HL3 solution containing 0.5 mM Ca^2+^. (D) Mean mEJP amplitude in *S6KL* mutants was significantly higher than wild types. *n* = 19, 23, and 20 recordings for wild type, *S6KL* ^*140*^, and neuronal rescue of *S6KL* ^*140*^, respectively. ***p<0.001 by one-way ANOVA with Tukey post hoc test; error bar indicates SEM. (E) Cumulative probability plot of mEJP amplitudes. The quantal size was shifted to the right in *S6KL* ^*140*^ mutants.

To examine the functional consequences of these altered NMJ synapses in *S6KL* mutants, we recorded both evoked excitatory junction potentials (EJPs) and spontaneous miniature EJPs (mEJPs) at muscle 6 using intracellular electrodes. The results showed that the mean EJP amplitude was not significantly altered in *S6KL* ^*140*^ mutants (39.32±1.586 mV for wild type and 41.66±1.249 mV for *S6KL* ^*140*^ mutants; *p* = 0.25). However, the mean mEJP amplitude, also known as quantal size, was 1.677±0.033 mV in *S6KL* ^*140*^ mutants, significantly higher than 1.123±0.021 mV in wild type (*p*<0.001; Fig. 6A, 6B, and 6D). A cumulative probability plot showed that 80% of mEJP amplitudes were < 1.5 mV in wild types, while only 47% of mEJP amplitudes were <1.5 mV in *S6KL* ^*140*^ mutants; the difference was significant by Kolmogorov-Smirnov test (*D* = 0.33; *p*<0.001; Fig. 6E). As with the restoration of normal NMJ morphology (Fig. 3), *elav-Gal4*-driven presynaptic expression of S6KL in *S6KL* ^*140*^ mutant background also fully rescued the increased mean mEJP amplitude (*p*>0.05 compared with wild type; Fig. 6C, 6D and 6E), indicating that the increased quantal size is specifically caused by *S6KL* mutations.

### Defective endocytosis at *S6KL* mutant NMJ synapses

**Fig 7:**
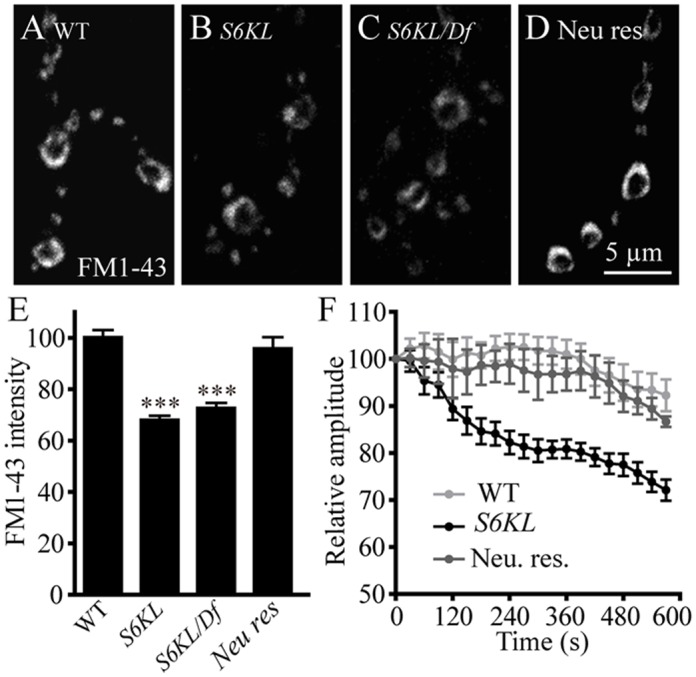
*S6KL* mutation causes reduced synaptic endocytosis and failure to sustain neurotransmission during high-frequency stimulation. (A–D) NMJ 4 synapses in abdominal segment A3 were loaded with FM1–43 in wild type (A), *S6KL* ^*140*^ (B), *S6KL* ^*140*^/*Df(1)ED7413* (C), and re-expression of S6KL driven by the motoneuron-specific *OK6-Gal4* in *S6KL* ^*140*^ background (Rescue) (D). Scale bar, 5 μm. (E) Quantification of FM1–43 fluorescence intensities in NMJ boutons following high K^+^-stimulated endocytosis. *n* = 14, 17, 10, and 19 NMJs for wild type, *S6KL* ^*140*^, *S6KL* ^*140*^ */Df(1)ED7413*, and rescue, respectively. ***p<0.001 by one-way ANOVA with Tukey post hoc test; error bars indicate SEM. (F) Average EJP amplitudes recorded during 10 Hz stimulation of motor axons for 10 min in 1 mM external Ca^2+^ in wild types, *S6KL* ^*140*^ mutants, and neuronal-rescued *S6KL* ^*140*^ mutants. *n* = 11, 11, and 8 recordings for the three genotypes, respectively. Average EJP amplitudes (binned per 30 s) were normalized to the initial response.

As with *S6KL* mutants, several endocytic protein mutants also show more satellite boutons, fewer and larger SVs, and increased mEJP amplitudes at NMJs [23,26–28]. To assess if endocytosis was affected by the *S6KL* mutation, we first performed FM1–43 dye uptake assays at the NMJs of third instar larvae. The lipophilic FM1–43 is non-fluorescent in the aqueous extracellular environment, but quantum yield is increased more than 40-fold when bound to lipid membrane. Hence, newly endocytosed vesicles in the presence of FM1–43 will be fluorescently labeled, providing a quantitative measure of vesicle endocytosis [8,29]. Compared with wild-type controls, which displayed intense labeling of FM1–43, dye uptake was reduced by 30.95% in *S6KL* ^*140*^ mutant boutons (*p*<0.001; Fig. 7A, 7B, and 7E). Similarly, hemizygous *S6KL* mutants (*S6KL* ^*140*^ */Df(1)ED7413*) also showed significantly reduced FM1–43 uptake compared with wild type (*p*<0.001; Fig. 7A, 7C, and 7E). The decrease in FM1–43 dye loading in *S6KL* mutants was completely reversed by presynaptic expression of *S6KL* driven by *OK6-Gal4* (Fig. 7A, 7D and 7E). These results suggest that *S6KL* is required for synaptic endocytosis.

Efficient endocytosis is required to sustain neurotransmitter release during intense stimulation. As shown in Fig. 7F, EJP amplitudes in *S6KL* mutants declined to about 72.13% of the initial response during 10 min tetanic stimulation at 10 Hz in 1 mM Ca^2+^. In contrast, wild-type controls maintained release at about 92.27% of the initial response after the same stimulus train. Again, this phenotype was fully rescued by presynaptic expression of S6KL in *S6KL* ^*140*^ mutants (Fig. 7F). The inability of *S6KL* mutants to maintain normal levels of transmission during intense activity is consistent with a defect in synaptic endocytosis or vesicle trafficking.

### S6KL interacts with BMP pathway components in regulating NMJ growth

**Fig 8:**
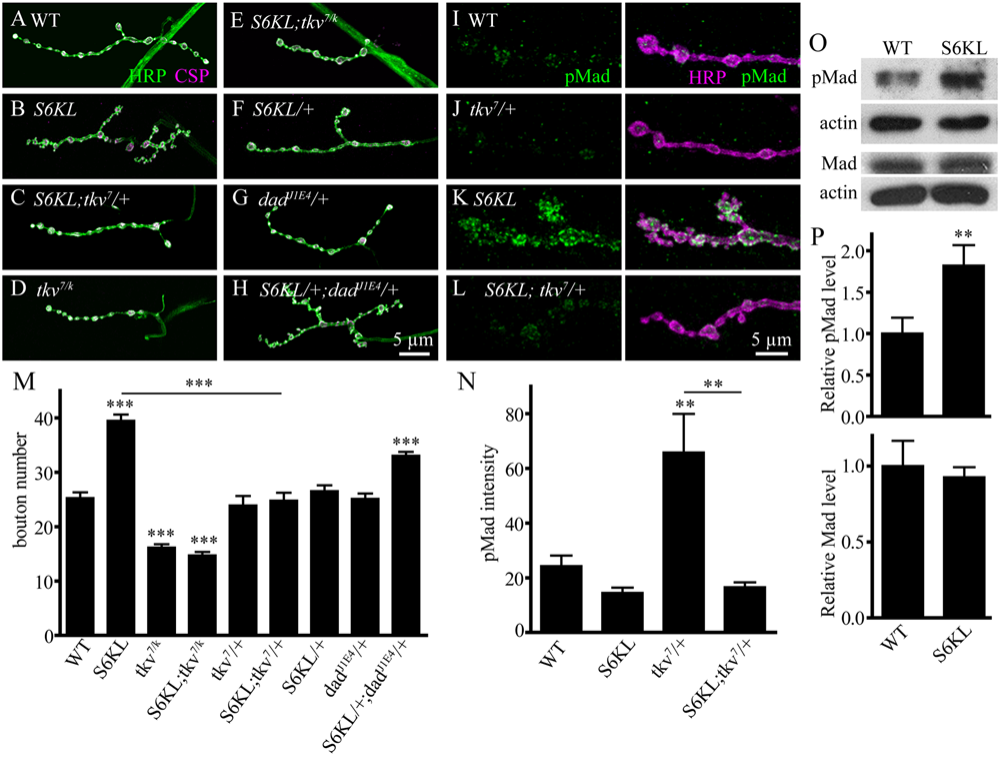
Genetic interactions between *S6KL* and components of the BMP signaling pathway in regulating NMJ growth. (A–H) Confocal images of NMJ 4 synapses of different genotypes co-stained with anti-HRP (green) and anti-CSP (magenta): WT (A), *S6KL* ^*140*^ (B), *S6KL* ^*140*^ *; tkv* ^*7*^ */+* (C), *tkv* ^*7*^ */tkv* ^*k16713*^ (D), *S6KL* ^*140*^ *; tkv* ^*7*^ */tkv* ^*k16713*^ (E), *S6KL* ^*140*^ */+* (F), *dad* ^*J1E4*^ */+* (G), and *S6KL* ^*140*^ */+; dad* ^*J1E4*^ */+* (H). Scale bar, 5 μm. (I–L) Confocal images of NMJ 4 terminals co-labeled with anti-pMad (green) and anti-HRP (magenta) in WT (I), *tkv* ^*7*^ */+* (J), *S6KL* ^*140*^ (K), and *S6KL* ^*140*^ *; tkv* ^*7*^ */+* (L) larvae. (M) Quantifications of synaptic bouton numbers in different genotypes including wild type (n = 17), *S6KL* ^*140*^ (n = 19), *tkv* ^*7*^ */tkv* ^*k16713*^ (n = 17), *S6KL* ^*140*^ *; tkv* ^*7*^ */tkv* ^*k16713*^ (n = 10), *tkv* ^*7*^ */+* (n = 16), *S6KL* ^*140*^ *; tkv* ^*7*^ */+* (n = 19), *S6KL* ^*140*^ */+* (n = 19), *dad* ^*J1E4*^ */+* (n = 10), and *S6KL* ^*140*^ */+; dad* ^*J1E4*^ */+* (n = 22). (N) Quantification of the relative fluorescence intensities of pMad in NMJ terminals of different genotypes. n = 8, 9, 8, and 8 for wild type, *tkv* ^*7*^ */+*, *S6KL* ^*140*^, and *S6KL* ^*140*^ *; tkv* ^*7*^ */+*, respectively. **p<0.01 by one-way ANOVA with Tukey post hoc test; error bars indicate SEM. (O) Western results of larval brains from wild-type control and *S6KL* ^*140*^ mutants. Actin was used as a loading control. (P) Quantification of the relative protein levels of pMad and Mad in the larval brains of wild type and *S6KL* ^*140*^ mutants. The level of pMad but not Mad was increased in *S6KL* mutants. *n* = 3, **p<0.01 by Student’s *t*-tests; error bars indicate SEM.

Up-regulation of BMP signaling leads to NMJ overgrowth characterized by more boutons including satellite boutons [14,16,17], similar to what we observed in *S6KL* mutants, suggesting that loss of *S6KL* may lead to upregulated BMP signaling. To examine possible involvement of BMP signaling in mediating the overgrowth of synaptic boutons in *S6KL* mutants, genetic interactions between *S6KL* and the BMP receptor *tkv* and other components of the pathway were examined. The NMJs in *tkv* ^*7*^ */tkv* ^*k16713*^ mutants were undergrown (25.24±1.11 boutons for WT and 16.13±0.66 boutons for *tkv* ^*7*^ */tkv* ^*k16713*^; *p*<0.001; Fig. 8A, 8D, and 8M). Interestingly, the number of total boutons in *S6KL* ^*140*^ *; tkv* ^*7*^ */tkv* ^*k16713*^ double mutants were not different from those in *tkv* single mutants (*p*>0.05; Fig. 8D, 8E, and 8M), suggesting that synaptic overgrowth induced by S6KL mutation requires BMP signaling through Tkv. Importantly, mutating one copy of the BMP type I receptor *tkv* in *S6KL* ^*140*^ background (*S6KL* ^*140*^ *; tkv* ^*7*^ */+*) completely reversed the increase in total bouton number in *S6KL* ^*140*^ mutants (24.80±1.46 boutons for *S6KL* ^*140*^ *; tkv* ^*7*^ */+* and 39.47±1.14 boutons for *S6KL* ^*140*^ mutants; *p*<0.001; Fig. 8A–8C and 8M), whereas the number of total boutons in heterozygous *tkv* ^*7*^ mutants (*tkv* ^*7*^ */+*) was normal as wild type (*p*>0.05; Fig. 8M). Similarly, mutating one copy of *mad*, which encodes the BMP effector mothers against decapentaplegic (25.20±0.92 boutons for *S6KL* ^*140*^ *; mad* ^*237*^ */+*; *p*<0.001 compared with *S6KL* ^*140*^ mutants), or *wit*, which encodes the type II BMP receptor Wit also suppressed the overgrown NMJs of *S6KL* mutants (23.47±1.22 boutons for *S6KL* ^*140*^ *; wit* ^*A12*^ */+*; *p*<0.001 compared with *S6KL* ^*140*^ mutants).

We then examined genetic interactions between *S6KL* and *dad*. Dad is a BMP signaling inhibitor and *dad* mutants show overgrown NMJ terminals [14,16]. Synaptic growth was normal in *S6KL* ^*140*^ */+* and *dad* ^*J1E4*^ */+* single heterozygous mutants. However, the total bouton number was 33.05±0.73 in *S6KL* ^*140*^ */+; dad* ^*J1E4*^ */+* transheterozygous mutants, significantly higher than that in the single heterozygotes (26.53±1.08 for *S6KL* ^*140*^ */+* and 25.13±0.97 for *dad* ^*J1E4*^ */+*; *p*<0.001; Fig. 8F–8H and 8M). These results together indicate that the excessive synaptic growth in *S6KL* mutants may be caused by up-regulation of BMP signaling.

Activation of BMP receptors by ligand binding leads to Mad phosphorylation (pMad), and pMad is the major transducer of BMP signaling in *Drosophila* motoneurons. Thus, pMad level serves as a molecular readout of BMP signaling. The intensity of pMad staining in the presynaptic terminals of *S6KL* ^*140*^ mutants was 2.71-fold higher than wild type (Fig. 8I, 8K, and 8N). Consistently, western analysis of larval brains showed an elevated level of pMad (1.82-fold higher than wild type) but a normal level of Mad protein in *S6KL* ^*140*^ mutants (Fig. 8O and 8P). Furthermore, the staining intensity of β–galactosidase encoded by *dad-lacZ* which has been widely used to monitor the transcriptional output of BMP pathway [30,31] was apparently increased in the motoneuron nuclei of *S6KL* mutants compared with wild type (S2 Fig.). As with the NMJ overgrown phenotype, pMad upregulation was reversed to wild-type level by mutating one copy of *tkv* in *S6KL* ^*140*^ background (Fig. 8I–8L and 8N). Taken together, these results indicate that the synaptic overgrowth in *S6KL* mutants requires BMP signaling and that S6KL normally serves to restrain NMJ growth by inhibiting BMP signaling.

### S6KL physically interacts with Tkv and negatively regulates its protein level

**Fig 9:**
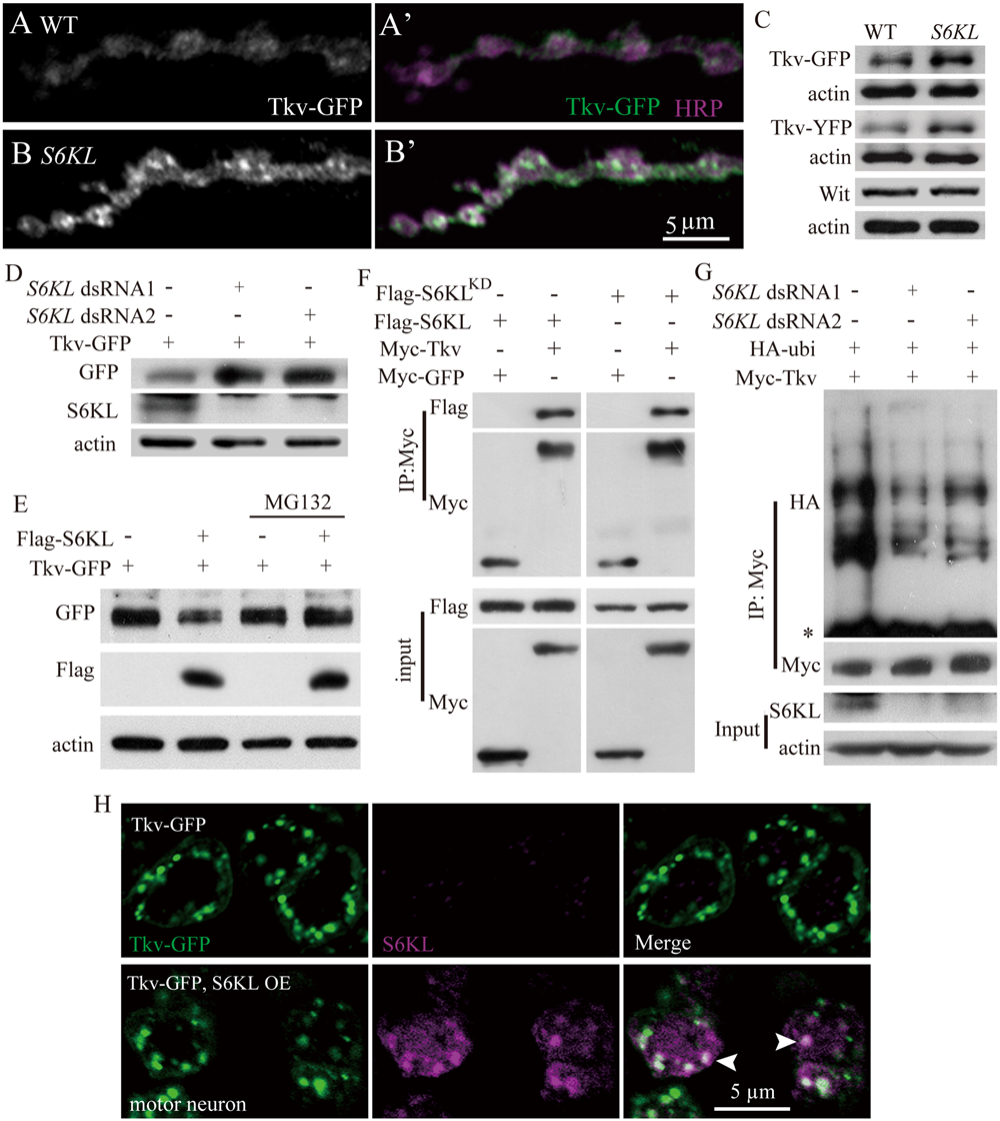
Negative regulation of Tkv protein level by S6KL via proteasomal degradation pathway. (A–B′) Confocal images of NMJ 4 branches double-labeled with anti-GFP (green) and anti-HRP (magenta) in control (*elav-Gal4/+; UAS-Tkv-GFP/+*) and mutants (*S6KL* ^*140*^ *elav-Gal4/S6KL* ^*140*^ *; UAS-Tkv-GFP/+*) showing elevated and punctate Tkv staining signals in *S6KL* ^*140*^ mutants. Scale bar, 5 μm. (C) Western results of total larval brain extracts probed with anti-GFP and anti-Wit antibodies. Actin was used as a loading control. (D) Tkv protein level was increased in S2 cells expressing a reduced level of S6KL. S2 cells were transfected with expression vector for Tkv-GFP and dsRNAs targeting two different sequences of the *S6KL* transcript. (E) Overexpression of S6KL suppresses Tkv protein level. S2 cells co-transfected with plasmids encoding Flag-S6KL and Tkv-GFP were untreated or treated with the proteasome inhibitor MG132 and subjected to western analysis with different antibodies. (F) Wild-type and kinase dead K193Q mutant (S6KL^KD^) S6KL interact with Tkv in S2 cells. S2 cells were co-transfected with expression vectors for Myc-Tkv or Myc-GFP and Flag-S6KL or Flag-S6KL^KD^. Cell lysates were subjected to immunoprecipitation with anti-IgG or anti-Flag and subsequently analyzed by western analysis. (G) Poly-ubiquitinated Tkv is decreased in S6KL—knockdown S2 cells. S2 cells were co-transfected with expression vectors for Myc-Tkv, HA-ubiquitin, and dsRNAs targeting two different sequences of the *S6KL* transcript. Asterisk denotes IgG. (H) S6KL and Tkv co-localize in the soma of motor neurons in the ventral nerve cord. Tkv-GFP alone (upper panels) or Tkv-GFP and S6KL (lower panels) were expressed under the control of the motoneuron specific *OK6-Gal4*. Scale bar, 5 μm.

To uncover the molecular mechanism underlying the up-regulation of BMP signaling in *S6KL* mutants, we examined the protein levels of two BMP receptors Tkv and Wit for which there are tags or antibodies are available. As a quality Tkv antibody was not available to us, we took advantage of the commercial GFP antibodies to detect the expression of GFP- or YFP-tagged Tkv. Immunostaining showed that the level of Tkv-GFP driven by the pan-neuronal *elav-Gal4* at NMJ terminals was higher in *S6KL* ^*140*^ mutants than wild types (Figs. 9A–9B’). Western analysis of larval brain extracts showed a 2.46 fold increase of Tkv-GFP protein level in *S6KL* ^*140*^ mutants than in wild types (Fig. 9C). Consistently, a 2.35 fold increase of Tkv protein level was detected by western analysis in *S6KL* ^*140*^ mutants expressing an YFP protein tag which labels the endogenous Tkv (Fig. 9C). In contrast, the protein level of Wit was not altered in *S6KL* ^*140*^ mutants compared with wild type (Fig. 9C). Quantitative PCR revealed that the level of *tkv* mRNA was normal in the larval brains of *S6KL* ^*140*^ mutants (S3 Fig.), indicating a negative regulation of Tkv by S6KL at the post-transcriptional level.

To further understand the specific effects of *S6KL* on Tkv protein expression, we first assessed Tkv-GFP protein levels in S2 cells expressing altered levels of S6KL by western analysis. The level of Tkv-GFP protein was higher in *S6KL* knockdown cells, whereas a decreased level of Tkv-GFP was observed in S6KL—overexpressing cells compared with control S2 cells expressing the endogenous level of S6KL (Fig. 9D and 9E). Furthermore, the reduction of the Tkv-GFP protein level in S6KL—overexpressing S2 cells was blocked by the potent 26S proteasome inhibitor MG132 (Fig. 9E), suggesting that S6KL down-regulates Tkv by facilitating proteasome-dependent degradation. To further confirm that S6KL affects Tkv protein level by proteasome pathway, we examined Tkv ubiquitination in S2 cells co-transfected with Myc-Tkv and double-stranded RNAs (dsRNA) against S6KL. In S6KL—knockdown cells with distinct dsRNAs, Tkv poly-ubiquitination was apparently decreased to different extents (Fig. 9G). We then performed immunoprecipitation assays from S2 cells co-expressing Flag-tagged S6KL and Myc-tagged Tkv or GFP to determine if S6KL physically interacted with Tkv. Western blotting of the immunoprecipitates by anti-Flag and anti-Myc antibodies revealed robust, specific co-immunoprecipitation of S6KL and Tkv (Fig. 9F), suggesting that S6KL forms a complex with Tkv in S2 cells, though an in vivo interaction between S6KL and Tkv remains to be established. The interaction was not dependent on the kinase activity, as the kinase dead S6KL^K193Q^ mutant still associated with Tkv (Fig. 9F), in support of a dominant negative effect of the point mutation [21,22]. Co-localization of overexpressed S6KL and Tkv-GFP in the soma of motor neurons in the ventral nerve cord (Fig. 9H) further supports the physical interaction between S6KL and Tkv.

## Discussion

In this study, we uncover that S6KL, a previously uncharacterized and evolutionally conserved serine-threonine kinase of the S6K family, inhibits NMJ growth by suppressing BMP signaling. We further demonstrate that S6KL suppresses BMP signaling by promoting proteasome-mediated degradation of the BMP receptor Tkv. Thus our results shed new light on the in vivo functions of S6KL and the regulation of BMP signaling in synapse development.

### S6KL plays a role distinct from S6K

S6K1 and S6K2 are downstream effectors of the mammalian target of rapamycin (mTOR) pathways [32]. S6K1 regulates cell growth by controlling the biosynthesis of translational components, most notably ribosomal proteins [32]. *S6K1* mutant mice are small in body size and exhibit glucose intolerance due to a selective decrease in β-cell size [33,34]. In *Drosophila*, there is only one S6K homologue named dS6K. *Drosophila dS6K* mutants show extreme delay in development and severe reduction in adult body size due to a reduced cell size of multiple cell types [35]. Several S6K substrates have been identified, the most extensively studied of which is ribosomal protein S6. In addition to S6, the eukaryotic translation initiation factor eIF4B is also a physiologically relevant target of S6K1 that could explain its effect on translation and cell growth; eIF4B is required for efficient recruitment of ribosomes to mRNA [36].

Different from *Drosophila dS6K* mutants, *S6KL* mutants showed normal body size and no delay in development, indicating that S6KL plays a role distinct from the cell growth regulation by S6K. At the NMJ terminals, S6KL also acts differently from dS6K. dS6K regulates bouton size, active zone number, and synaptic function without influencing bouton number [37,38], whereas S6KL inhibits NMJ growth by attenuating BMP signaling. The in vivo functions of S6KL in other organisms such as *C*. *elegans* and humans remain to be characterized.

### S6KL restrains NMJ growth by inhibiting BMP signaling

Bone morphogenetic protein signaling is the prevalent retrograde pathway promoting *Drosophila* NMJ growth [2,4,5,8]. In this study, we present genetic and immunochemical data demonstrating that S6KL restrains synapse growth by antagonizing BMP signaling activity. First, the NMJ terminals were overgrown with more synaptic boutons and satellite boutons in *S6KL* mutants (Fig. 3), consistent with enhanced BMP signaling. Second, the NMJ overgrowth phenotype was reversed by removing one copy of the BMP signaling pathway components such as *tkv* (Fig. 8C and 8M). Third, *S6KL* and *dad* mutants displayed dosage-sensitive genetic interactions; trans-heterozygotes of *S6KL* and *dad* had significantly more boutons compared with that of heterozygous mutants for either gene alone (Fig. 8H and 8M). This type of genetic interaction is rarely observed for gene products that do not directly influence each other. Finally, the immunostaining intensity of pMad and dad-LacZ, two distinct reporters of BMP signaling strength, was increased in *S6KL* mutant NMJs (Fig. 8K and 8N; S2 Fig.). Together, our data demonstrate that S6KL normally inhibits NMJ growth by antagonizing BMP signaling.

In addition to the up-regulation of BMP signaling in *S6KL* mutants, synaptic endocytosis is impaired in *S6KL* mutants. As endocytosis attenuates BMP signaling [14], one simple explanation for the elevated BMP signaling in *S6KL* mutants is defective endocytosis. However, our results suggest that S6KL may not directly affect endocytosis in regulating NMJ growth. First, synaptic proteins, such as Endophilin, Dynamin, and Eps15 are normally expressed at *S6KL* mutant NMJs (S4 Fig.), indicating that S6KL does not affect the protein level and localization of these proteins at NMJ synapses. In contrast, many endocytic proteins such as Dynamin, Synaptojanin, AP180, and Endophilin are reduced or mislocalized at endocytic mutant NMJs [24,28,39]. Second, the protein level of Tkv but not Wit was specifically increased in *S6KL* mutants compared with wild type, while both Tkv and Wit protein level was normal in *endoA* mutants (S5 Fig.). Immunostaining further confirmed an elevated level of Tkv in *S6KL* mutants but a normal level of Tkv in endocytic mutants (Fig. 8 and S6 Fig.). Similarly, a normal level of BMP receptors was reported in *spichthyin* and *nervous wreck* mutants with endocytic trafficking defects [14,17]. Thus, although *S6KL* mutants share key synaptic phenotypes with endocytic mutants such as satellite boutons, reduced FM1–43 uptake, more large SVs, increased amplitudes of mEJPs, and faster decline of EJP amplitudes (Figs. 3–7) [8,19,23,26,27,39,40], S6KL negatively regulates Tkv protein level whereas endocytic proteins do not.

There are multiple possibilities, not mutually exclusive, for the observed endocytic defects in *S6KL* mutants. First, the increased level of Tkv proteins in *S6KL* mutants may impinge on endocytosis through endocytic proteins, as Tkv interacts with Dap160 and Dynamin via nervous wreck [14]. Second, while endocytosis inhibits BMP signaling [14], BMP signaling may in turn suppress endocytosis. For example, increased BMP signaling in *dad* mutants results in endocytosis defects [8]. In addition, the endocytic defect detected by FM1–43 uptake in both *S6KL* and *Brat* mutants could be rescued by reducing the dose of BMP signaling components (S7 Fig. and [8]). Trio, a Rac GTPase-specific guanine nucleotide exchange factor, is a downstream target of BMP signaling in motoneurons [41]. Thus, the compromised endocytosis in *S6KL* mutants could be attributable, at least partially, to elevated BMP signaling and subsequently enhanced Trio-Rac-mediated actin reorganization, as actin cytoskeleton is closely involved in endocytosis [19]. Last, but not least, S6KL may affect endocytosis by directly regulating the activity, localization, or both of an endocytic protein not examined in this study. The exact mechanism by which loss of S6KL leads to endocytic defects remains to be characterized.

### S6KL facilitates proteasome-mediated degradation of Tkv

Multiple lines of evidence support that S6KL physically interacts with Tkv, and facilitates its proteasomal degradation. First, Tkv functions downstream of S6KL and is required for synaptic overgrowth in *S6KL* mutants (Fig. 8). Second, Tkv protein level was elevated in *S6KL* mutants and S2 cells expressing reduced levels of S6KL by RNAi knockdown (Fig. 9C and 9D). Conversely, Tkv protein level was decreased in S6KL—overexpressing S2 cells, and this decrease was blocked by MG132, a potent 26S proteasome inhibitor (Fig. 9E). The negative regulation of Tkv by S6KL is specific, as the protein levels of Wit and Mad, two components of the BMP signaling, remain unaltered in *S6KL* mutants (Figs. 8 and 9). Third, S6KL can be co-immunoprecipitated with Tkv from S2 cells and co-localizes with Tkv in the motoneuron soma (Fig. 9F and 9H), suggesting that they interact physically, though an in vivo interaction between the two remains to be determined. Together, our results show that in the absence of S6KL, Tkv accumulates, leading to upregulated BMP signaling and consequently overgrowth of NMJ terminals and defective endocytosis. It is worth noting that the negative regulation of BMP signaling by S6KL also applies to vein development in the wing (S8 Fig.).

How might S6KL target Tkv for degradation? The strong biochemical and genetic interactions between S6KL and Tkv raised several possible models for S6KL to inhibit Tkv degradation. The simplest model is that S6KL directly phosphorylates Tkv and facilitates its proteasomal degradation. However, we found no phosphorylation of Tkv by S6KL in an in vitro kinase assay. Alternatively, S6KL could act as an adaptor to recruit critical proteins, e.g., unidentified kinases and ubiquitin E3 ligases, initiating proteasomal degradation of Tkv, as has been reported for transmembrane receptors in different processes [31,42,43]. For example, Fused, a serine/threonine kinase, functions in concert with the E3 ligase Smurf to regulate ubiquitination and proteolysis of Tkv in the cystoblasts of the *Drosophila* female germline [31]. Fused likely acts on Tkv by phosphorylating the Ser238 site of Tkv, leading to Tkv ubiquitination by Smurf and subsequent proteasome-mediated degradation, though experimental evidence demonstrating Tkv is a direct phosphorylation target of Fused is still lacking [31]. In the *Drosophila* nervous system, S6KL may exert a function similar to Fused in the ovary, inhibiting BMP signaling by promoting Tkv degradation. As *Smurf* null mutants showed normal NMJ terminals (S9 Fig.), the identity of the ubiquitin ligase that cooperates with S6KL in motoneurons to promote Tkv degradation remains to be determined. In addition, as membrane receptors can be degraded by lysosomes [43,44], S6KL facilitated degradation of Tkv by lysosomes is also possible.

Regardless of detailed mechanisms, our study has established a close connection between S6KL and Tkv, and uncovered a critical role of S6KL in regulating synaptic growth and function by inhibiting BMP signaling activity through promoting Tkv degradation. Because of the broad roles of tightly regulated BMP signaling in development and dysregulation of BMP signaling in many diseases, including cancer and nervous system diseases, it will be of great interest to determine if mammalian homologs of S6KL act similarly on BMP signaling.

## Materials and Methods

### *Drosophila* strains and genetics

Fly stocks were maintained on standard cornmeal food at 25°C unless specified. The *w* ^*1118*^ strain was used as the wild type control in all experiments. *P* element-mediated excisions were used to generate deletions in *S6KL* following a standard protocol. The original stock *EY10051* from the Bloomington Stock Center has a *P* element insertion in the first intron of *S6KL* (Fig. 1D). Before mobilizing *EY10051* by *P* transposase Δ2–3, we isogenized the original stock. The *w* ^+^ deletion lines with the *P* insertion excised imprecisely (*S6KL* ^*158*^ *and S6KL* ^*140*^) were verified by PCR, DNA sequencing, and western blotting.

Transgenic lines carrying *UAS-S6KL* or *UAS-S6KL* ^*K193Q*^ (a critical ATP binding residue lysine at 193 mutated to glutamine) were generated by inserting the full-length open reading frame of wild-type or mutant *S6KL* into the *pUAST* vector, followed by standard germline transformation. The tissue-specific Gal4 drivers *elav-Gal4* (pan-neuronal), *OK6-Gal4* (motor neuron specific), *Mhc-Gal4* (muscle specific), and *C57-Gal4* (muscle specific) were used for tissue-specific expression experiments. The *UAS-Tkv-GFP* transgenic line was supplied by Marcos Gonzalez-Gaitan (University of Geneva, Geneva, Switzerland). A yellow fluorescent protein (YFP) genetrap line labeling the endogenous Tkv with YFP was obtained from the *Drosophila* Genome Research Center (stock number 115298; Kyoto, Japan). The *S6KL* RNAi lines 102179 and 47326 were from the Vienna *Drosophila* Stock Center (VDRC). The following stocks were obtained from the Bloomington Stock Center: *EP438*, *Df(1)ED7413*, *wit* ^*A12*^, *wit* ^*B11*^, *tkv* ^*k16713*^, *tkv* ^*7*^, *dad* ^*j1E4*^, and *mad* ^*237*^.

Information on additional stocks, as well as antibodies and quantitative PCR analysis, can be found in S1 Text.

### Generation of rabbit antibodies against S6KL

Polyclonal antibodies against S6KL were raised by immunizing rabbits with peptides containing amino acid residues 66–82 (TNQPTQSHPPGDEEQPV) of S6KL. The antibodies were purified using an affinity column coupled with the S6KL peptide according to the manufacturer’s instructions and used at 1:1000 for both staining and western analysis.

### Immunoprecipitation and in vitro kinase activity assay

Coding sequences for GFP, S6KL, and S6KL^K193Q^ were subcloned into pAC5.1 plasmids to produce N-terminally Flag tagged fusion proteins. Recombinant plasmids were transfected into S2 cells by Cellfectin II reagent (Invitrogen). After 48 h, transfected cells were lysed in RIPA buffer (50 mM Tris-HCl at pH 7.4, 150 mM NaCl, 0.1% SDS, 1% NP-40) for 1 h at 4°C. Cell lysates were precleared with protein G-Sepharose (GE) for 1 h and then incubated with anti-Flag monoclonal antibody (Sigma) for 3 h at 4°C. The immuno-complexes were precipitated with protein G-sepharose (GE), washed five times with RIPA buffer, and then washed two times with kinase buffer containing 25 mM Tris-HCl (pH 7.5), 5 mM β-glycerophosphate, 2 mM dithiothreitol (DTT), 0.1 mM Na_3_VO_4_, and 10 mM MgCl_2_. To perform the kinase activity assay, the anti-Flag immunoprecipitates were incubated with 50 μM ATP (Sigma), 10 μCi [γ-^32^P] ATP (PerkinElmer) and 5 μg myelin basic protein (Sigma) as substrates for 40 min at 30°C. The reaction products were separated on 12% SDS-polyacrylamide gels and autoradiographed to detect γ-^32^P incorporation.

### Immunohistochemistry and confocal microscopy

Immunostaining of larval fillets was performed as described with minor modifications [7,8]. Late third instar larvae were dissected in Ca^2+^-free standard saline, fixed in fresh 4% paraformaldehyde for 40 min, and then immunostained with the following primary antibodies: FITC-conjugated goat anti-HRP at 1:100 (Jackson ImmunoResearch), rabbit anti-S6KL at 1:1000, rabbit anti-phosphorylated Mad at 1:500 (from P. ten Dijke, Leiden University, Leiden, the Netherlands) [45], and anti-CSP at 1:500 (6D6, Developmental Studies Hybridoma Bank, DSHB). Alexa 488- or 568-conjugated anti-mouse or anti-rabbit secondary antibodies (Invitrogen) were used at 1:1000 to visualize primary antibody staining. All images were acquired using a Leica SP5 or Olympus BX51 laser scanning confocal microscope and processed with Adobe Photoshop.

Morphological analysis of NMJs was described previously [7,8]. Briefly, serial confocal images of NMJ 4 in the abdominal segments A2 and A3 were acquired and individual boutons were identified by immunostaining with the synaptic vesicle marker anti-CSP.

### Electron microscopy

Electron microscopy of NMJ synapses was performed as previously described [8,19]. Briefly, dissected larvae were fixed overnight at 4°C in a mixture of 2% glutaraldehyde and 2% paraformaldehyde in 0.1 M cacodylate buffer (pH 7.4), rinsed in cacodylate buffer, and post-fixed in 0.5% OsO_4_/0.8% K_3_Fe(CN)_6_ cacodylate buffer for 90 min at room temperature. Samples were washed and stained in saturated aqueous uranyl acetate for 2 h on ice, dehydrated in a graded acetone series, and embedded in Spurr resin (Electron Microscopy Sciences). Ultrathin (70–80 nm) longitudinal sections of NMJ6/7 were cut with a Leica UC6 ultramicrotome. Grids were post-stained with saturated uranyl acetate for 20 min, followed by staining with Reynold’s lead citrate for 2–3 min. Processed samples were observed under a JEOL JEM-1400 transmission electron microscope. Statistical analysis of synaptic vesicles was performed as previously described [8,19].

### Electrophysiology

Intracellular recordings from *Drosophila* muscle were performed essentially as described [8,19]. Briefly, wandering third instar larvae were dissected in calcium free HL3 saline [46] (in mM, 70 NaCl, 5 KCl, 20 MgCl_2_, 10 NaHCO_3_, 5 trehalose, 5 HEPES, 115 sucrose; pH 7.3). Excitatory junctional potentials (EJPs) were evoked at 0.3 Hz by a suction electrode using a depolarizing pulse delivered by a Grass S48 stimulator (Astro-Grass Inc.). Both EJPs and miniature EJPs (mEJPs) were recorded from muscle 6 of abdominal segment A2 or A3 in HL3 saline containing 0.5 mM Ca^2+^ using intracellular microelectrodes (10–20 MΩ) filled with 3 M KCl, and processed with Clampfit 10.2 software. To examine synaptic transmission upon high-frequency stimulation, presynaptic axons were stimulated at 10 Hz for 10 min in HL3 saline containing 1 mM extracellular Ca^2+^ [19,47]. All electrophysiological responses were recorded using Axoclamp 2B amplifier (Axon Instruments) in Bridge mode and analyzed using Clampfit 10.2 software. Only recordings from cells with resting potentials ≤ -60 mV and input resistances > 6 MΩ were analyzed.

### FM1–43 uptake assay

Fluorescent lipophilic FM dyes are widely used to follow endocytosis, vesicle trafficking, and exocytosis. The FM1–43 uptake assay for measurement of endocytosis at NMJ synapses was described previously [8,29]. Briefly, late third instar larvae were dissected in low Ca^2+^ (0.2 mM) saline while keeping the central nervous system intact and then perfused with normal medium containing (in mM) 128 NaCl, 2 KCl, 4 MgCl_2_, 1.8 CaCl_2_, 5 HEPES and 35.5 sucrose at pH 7.3 [48]. Endocytosis of FM1–43 was induced by high K^+^ (90 mM) in the normal medium containing 10 μM FM1–43 (Molecular Probes) and reduced NaCl by an equivalent amount for 3 min. Depolarized and FM1–43-loaded preparations were then vigorously washed three times with Ca^2+^-free saline (normal medium with 0 Ca^2+^ and 0.5 mM EGTA) for 2 min and imaged using a Leica SP5 confocal microscope with a 40× water-immersion lens for multiple Z stack sections.

### Cell culture, co-immunoprecipitation, and western analysis

Culture of *Drosophila* S2 cells and RNAi-mediated knockdown of gene expression were performed using protocols previously described [49]. S2 cells were maintained at 25°C in optimized serum-free medium (SF-900 II, Gibco). Cells were transfected using Cellfectin II reagent (Invitrogen) and expression was induced with 0.5 mM CuSO_4_ for 48 h. For dsRNA synthesis, the DNA template was PCR-amplified from a plasmid containing the full-length *S6KL* ORF using the following *S6KL—*specific primer pairs, 5’-TAATACGACTCACTATAGGGGCAGCAGGAGAATCCAGTTC-3’ and 5’-TAATACGACTCACTATAGGGTTGTGGAGAAAATCCAAGGC-3’ for S6KL dsRNA1, and 5’-TAATACGACTCACTATAGGGGAAGCAAATAGATCTGTGGCAAAAC-3’ and 5’-TAATACGACTCACTATAGGGGCTGCTTGGAGCTCAGGTTATAGTC-3’ for S6KL dsRNA2. The PCR products were used as templates for synthesis of double-stranded RNA using the MEGAscript T7 kit (Ambion). For RNAi-induced gene silencing, cultured cells were resuspended at a final concentration of 1×10^6^ cells per ml medium and plated at 1 ml per well in six-well culture plates. After 24 h incubation, 10 μg dsRNA was added and mixed by gently swaying the plate in a circular motion six to eight times. Forty eight hours later, transfected cells were lysed in RIPA buffer for 1 h at 4°C. Protein expression levels were analyzed using the following antibodies: rabbit anti-S6KL at 1:1000, anti-GFP at 1:1000 (Clontech), anti-Flag at 1:1000 (Sigma), and anti-actin at 1:50,000 (Millipore). For immunoprecipitation, S6KL—Flag and Tkv-Myc were co-expressed in S2 cells. Cell lysates were pre-cleared with protein G-Sepharose (GE) for 1 h and then incubated with anti-Flag antibody at 1:100 (Sigma) or anti-Myc antibody at 1:100 (Millipore) for 3 h at 4°C. The immune complexes were precipitated with protein G-sepharose (GE), which were then washed five times with ice-cold RIPA buffer and boiled for 5 min in 30 μl sample buffer. Freed proteins were then separated by SDS-PAGE and subjected to western analysis using anti-Flag at 1:1000 and anti-Myc at 1:1000. The secondary antibody was HRP-coupled anti-mouse IgG (Sigma) or anti-rabbit IgG (Sigma) used at 1:50000. The protein bands were visualized using a chemiluminescent HRP substrate (Millipore).

To determine whether the degradation of Tkv was dependent on proteasome, S2 cells were transfected with plasmids co-expressing Flag-S6KL and Tkv-GFP. After 48 h culture, cells were treated with or without 50 μM MG132 (Sigma) for additional 4 h, followed by western analysis.

For western blotting of different genotypes, 60 adult heads from each genotype were homogenized in ice-cold 120 μl RIPA lysis buffer containing 2% SDS. The immunoblots (proteins from ten heads of each genotype per lane) were incubated overnight at 4°C with anti-S6KL at 1:1000, anti-GFP at 1:1000, and anti-Wit at 1:50 (DSHB). Actin was used as a loading control.

### Statistical analysis

Quantification of pMad staining and endocytosed FM1–43 intensities was performed using Image J. Anti-HRP staining was used as an internal control for pMad intensity quantification. For each channel, an arbitrary threshold was set and used for all relevant images. Each staining experiment was repeated at least three times. To quantify the expression levels of target proteins, positive signals on western blots from multiple independent repeats were calculated using ImageJ and normalized to the actin control. All the data are expressed as mean ± standard error of the mean (SEM). Statistical significance between paired group means was determined by Student’s *t*-tests (Fig. 5). Multiple group means were evaluated by one-way ANOVA with Tukey’s *post hoc* tests for pair-wise comparisons. The cumulative probabilities of quantal size between wild type and *S6KL* mutants in Fig. 6E were compared by Kolmogorov-Smirnov test. All comparisons were between a specific genotype and the control unless otherwise indicated. No asterisk denotes *p*>0.05, * *p*<0.05, ** *p*<0.01, and *** *p*<0.001.

## Supporting Information

S1 Fig S6KL is not expressed in wing and eye discs, but expressed in a small population of cells in leg disc. A–E, Representative staining results of a wing discs from wild type (A), *S6KL* ^*140*^ (B), and *Vg-Gal4/+; UAS-S6KL/+* (C), and a leg disc from wild type (D) and *S6KL* ^*140*^ (E) double-stained with anti-S6KL (red) and TO-PRO-3 (labeling nuclei; green). S6KL driven by Vg-Gal4 is expressed in the presumptive wing blade and along dorsal/ventral compartment boundary (arrow) in wing disc. Arrows in D indicate S6KL predominantly expressed in a small population of cells in the center of leg disc which develops into distal claw. Scale bar, 40 μm. F and G, Representative staining results of eye discs double-stained with anti-S6KL and anti-HRP from wild type (F) and *S6KL* ^*140*^ (G). Scale bar, 10 μm.
(TIF)

S2 Fig Upregulation of *dad-lacZ* expression in the motoneuron nuclei of *S6KL* mutant ventral nerve cords. Representative projected confocal images of ventral nerve cords in *dad-lacZ/+* control (A), *S6KL* ^*140*^ *; dad-lacZ/+* (B), and *tkv* ^*7/k16713*^ *; dad-lacZ/+* (C) stained with anti-β-Gal. Scale bar, 10 μm. Motoneurons are indicated by GFP under the control of the motoneuron specific *OK6-Gal4* (D).
(TIF)

S3 Fig The mRNA level of *tkv* was not significantly changed in *S6KL* mutants. The *tkv* mRNA level normalized to the actin mRNA level in the larval brains of wild type and *S6KL* mutants. No significant difference in *tkv* mRNA levels between the two genotypes by Student’s *t*-tests. *n* = 4, error bars indicate SEM.
(TIF)

S4 Fig Endocytic proteins localize normally in *S6KL* mutant NMJ synapses. Representative confocal images of NMJ 4 synapse in wild type (A, C, and E) and *S6KL* ^*140*^ mutants (B, D, and F) labeled with anti-Endophilin A (A and B), anti-Dynamin (C and D), and anti-Eps15 (E and F). Scale bar, 2 μm.
(TIF)

S5 Fig The endogenous Tkv protein level is obviously increased in *S6KL* but normal in *endoA* mutants. (A) Western results of larval brains from wild type, *S6KL* ^*140*^, and *endoA* ^*Δ4*^ */endoA* ^*EY02730*^ mutants probed with anti-Tkv (recognizing multiple Tkv isoforms) and anti-Wit antibodies. The specificity of anti-Tkv was verified in *tkv* ^*7*^/*tkv* ^*k16713*^ mutants. Actin was used as a loading control. (B and C) Quantification of the relative protein levels of Tkv (B) and Wit (C) in the larval brains of wild type, *S6KL* ^*140*^, and *endoA* ^*Δ4*^ */endoA* ^*EY02730*^ mutants. The level of Tkv was increased in *S6KL* but not *endoA* mutants (B); the level of Wit was unaltered in both mutants (C). *n* = 3, **p<0.01 by Student’s *t*-tests; error bars indicate SEM.
(TIF)

S6 Fig Tkv-GFP staining pattern at NMJ synapses is not altered in *synj* mutants. Confocal images of NMJ 4 synapses double-labeled with anti-GFP (green) and anti-HRP (magenta) in control (*elav-Gal4/+;+/+;UAS-Tkv-GFP/+*) (A) and *synaptojanin* mutants (*elav-Gal4/+;synj* ^*1*^ */synj* ^*Ly*^ *;UAS-Tkv-GFP/+*) (B) showing similar Tkv-GFP staining signals. Arrowheads indicate satellite boutons. Scale bar, 5 μm.
(TIF)

S7 Fig Decreased FM1–43 uptake in *S6KL* mutants is fully rescued by reducing the dose of *tkv* by half. (A–D) NMJ 4 synapses in abdominal segment A3 were loaded with FM1–43 in wild type (A), *S6KL* ^*140*^ (B), *tkv* ^*7*^/+ (C), and *S6KL* ^*140*^ *;tkv* ^*7*^ */+* (D). Scale bar, 5 μm. (E) Quantification of FM1–43 fluorescence intensities in NMJ boutons following high K^+^-stimulated endocytosis. *n* = 29, 20, 26, and 25 NMJs for wild type, *S6KL* ^*140*^, *tkv* ^*7*^/+, and *S6KL* ^*140*^ *;tkv* ^*7*^ */+*, respectively. ***p<0.001 by one-way ANOVA with Tukey post hoc test; error bars indicate SEM.
(TIF)

S8 Fig Negative regulation of BMP signaling by S6KL in the wing. Whole wing images from different genotypes are shown (A-J). *dadJ^1E4^*, *smurf15C*, and *S6KL^140^* mutants showed normal vein pattern and wing morphology. However, overexpression of *S6KL* throughout wing blade driven by *MS1096-Gal4* led to the absence of the anterior cross vein (ACV, indicated by arrows), recapitulating that of *tkv* mutants (compare B and F). Overexpression of *S6KL* rescued the ectopic vein phenotypes (indicated by white arrowheads in G) caused by *Mad* overexpression (compare G and H), but did not rescue the wing phenotype caused by *Tkv-GFP* overexpression, presumably due to its strong effect (compare I and J).
(TIF)

S9 Fig Normal NMJ growth in *smurf* mutants. (A–E) Representative NMJ 4 synapses of different genotypes double-stained with anti-HRP recognizing neuronal plasma membrane (green) and an antibody against CSP (magenta), a synaptic vesicle protein. The genotypes are: WT (A), *Df(2R)Exel7149/+* (B), *smurf* ^*15c*^ */+* (C), hemizygous *smurf* ^*15c*^ */Df(2R)Exel7149* (D), and *elav/+; Smurf RNAi/+* (E). Scale bar, 5 μm. (F) Statistical results of the number of total boutons in different genotypes. *n* = 18, 12, 16, 30 and 13 NMJs for WT, *Df(2R)Exel7149/+*, *smurf* ^*15c*^ */+*, *smurf* ^*15c*^ */Df(2R)Exel7149*, and *elav/+; Smurf RNAi/+*, respectively. Error bars indicate SEM.
(TIF)

S1 Text Detailed information on additional *Drosophila* stocks, antibodies, and quantitative PCR analysis. (DOCX)
