# Supplementary figures and images for: Drosophila S6 Kinase Like Inhibits Neuromuscular Junction Growth by Downregulating the BMP Receptor Thickveins
Source: PLoS Genet. 2015 Mar 6;11(3):e1004984. doi: 10.1371/journal.pgen.1004984 (PMC4351882; doi:10.1371/journal.pgen.1004984)

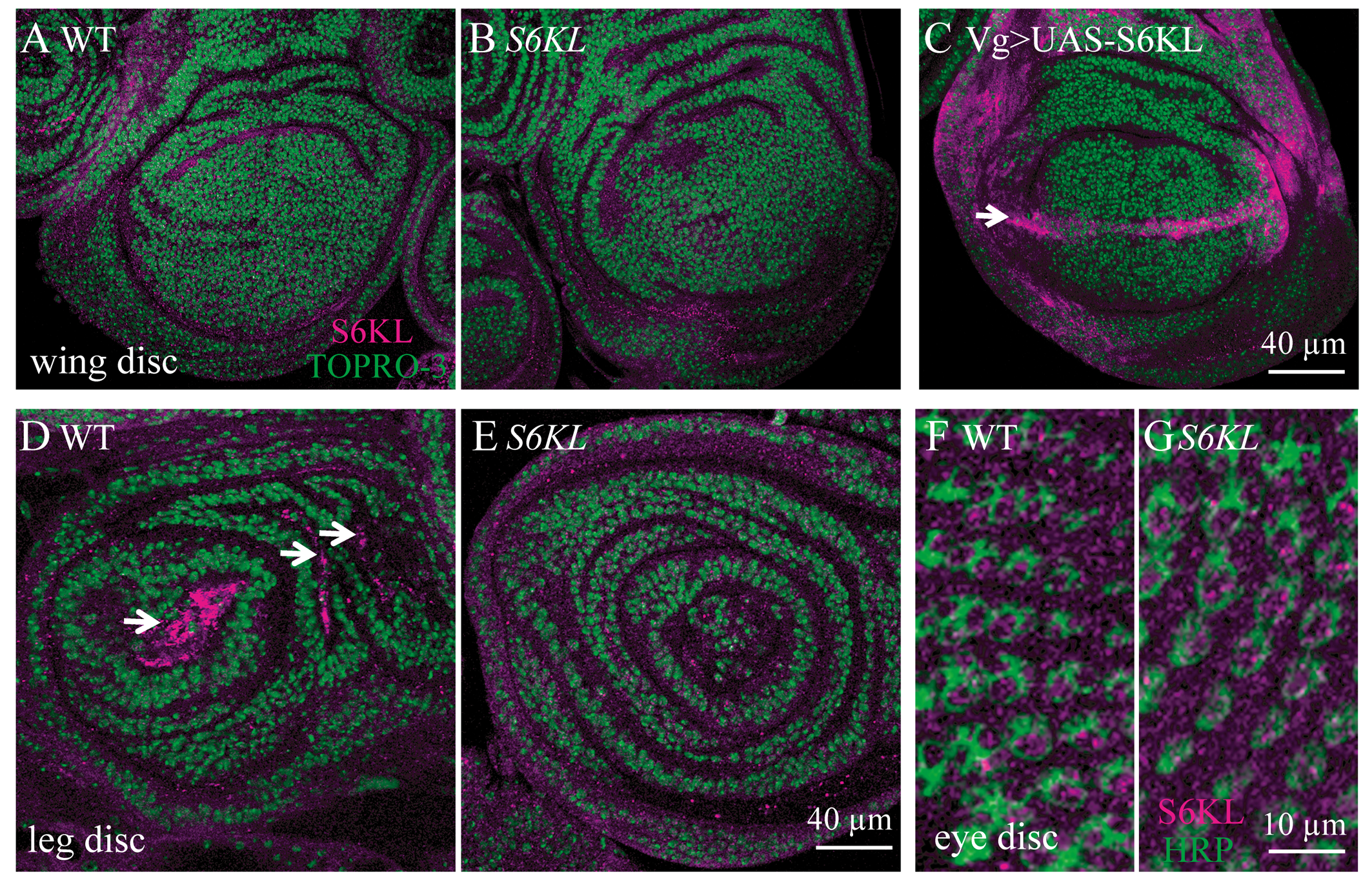

Supplement: S1 Fig — A–E, Representative staining results of a wing discs from wild type (A), S6KL 140 (B), and Vg-Gal4/+; UAS-S6KL/+ (C), and a leg disc from wild type (D) and S6KL 140 (E) double-stained with anti-S6KL (red) and TO-PRO-3 (labeling nuclei; green). S6KL driven by Vg-Gal4 is expressed in the presumptive wing blade and along dorsal/ventral compartment boundary (arrow) in wing disc. Arrows in D indicate S6KL predominantly expressed in a small population of cells in the center of leg disc which develops into distal claw. Scale bar, 40 μm. F and G, Representative staining results of eye discs double-stained with anti-S6KL and anti-HRP from wild type (F) and S6KL 140 (G). Scale bar, 10 μm. (TIF) [file pgen.1004984.s001.tif]

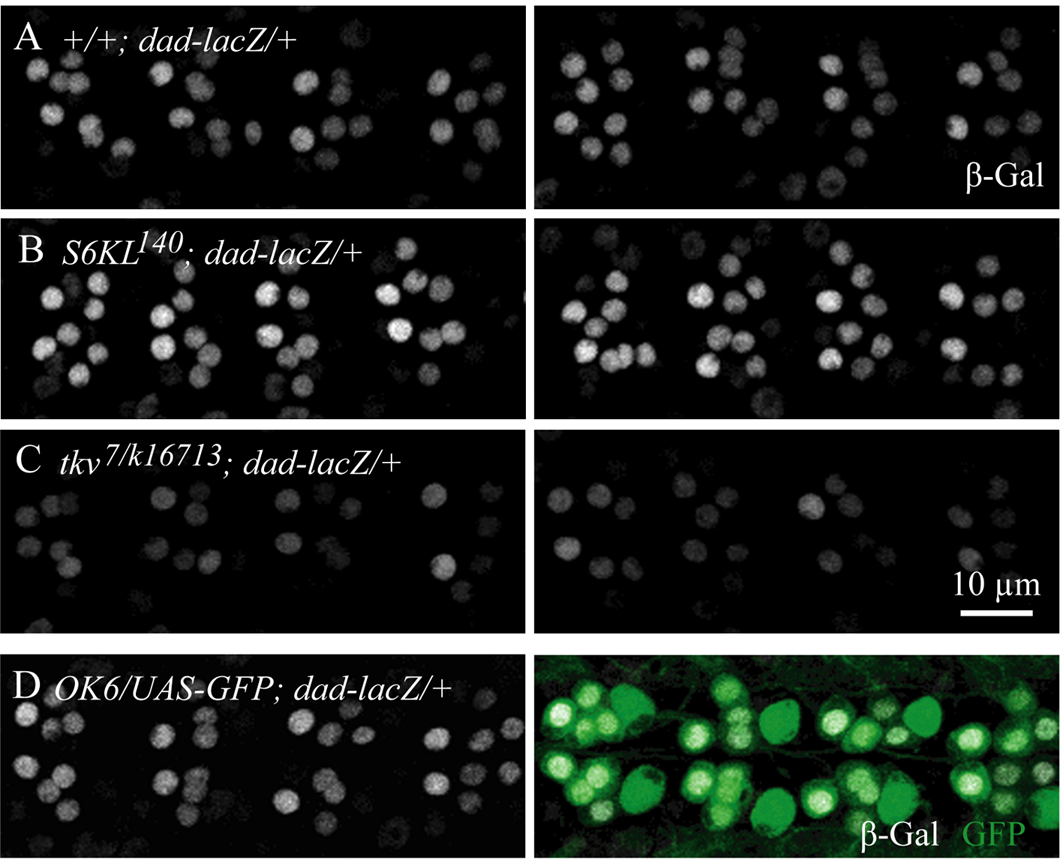

Supplement: S2 Fig — Representative projected confocal images of ventral nerve cords in dad-lacZ/+ control (A), S6KL 140 ; dad-lacZ/+ (B), and tkv 7/k16713 ; dad-lacZ/+ (C) stained with anti-β-Gal. Scale bar, 10 μm. Motoneurons are indicated by GFP under the control of the motoneuron specific OK6-Gal4 (D). (TIF) [file pgen.1004984.s002.tif]

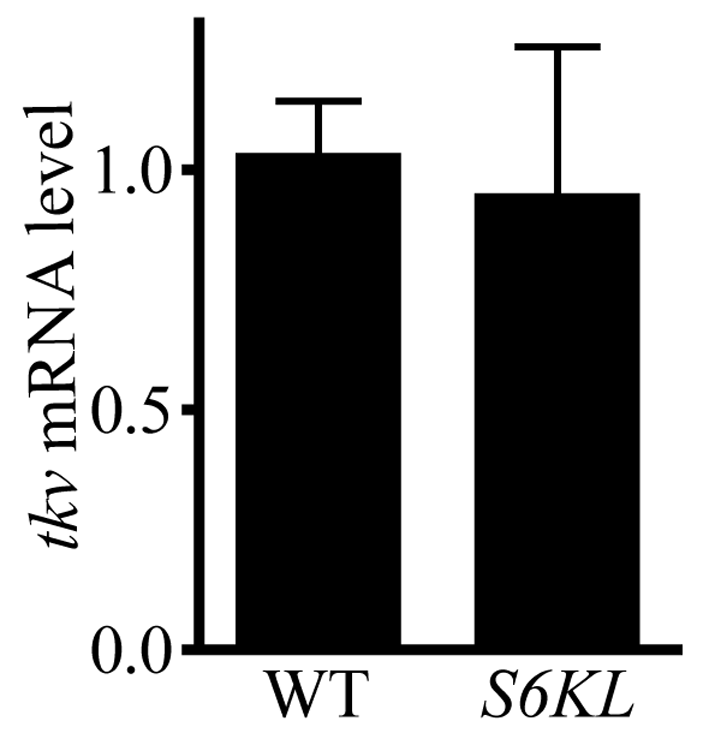

Supplement: S3 Fig — The tkv mRNA level normalized to the actin mRNA level in the larval brains of wild type and S6KL mutants. No significant difference in tkv mRNA levels between the two genotypes by Student’s t-tests. n = 4, error bars indicate SEM. (TIF) [file pgen.1004984.s003.tif]

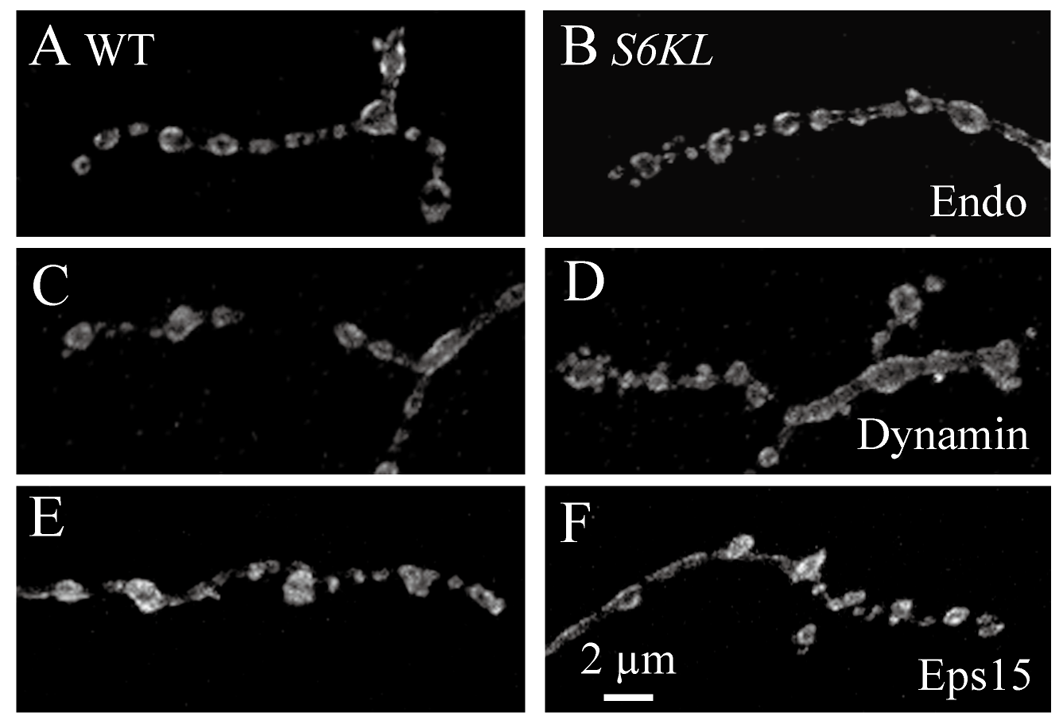

Supplement: S4 Fig — Representative confocal images of NMJ 4 synapse in wild type (A, C, and E) and S6KL 140 mutants (B, D, and F) labeled with anti-Endophilin A (A and B), anti-Dynamin (C and D), and anti-Eps15 (E and F). Scale bar, 2 μm. (TIF) [file pgen.1004984.s004.tif]

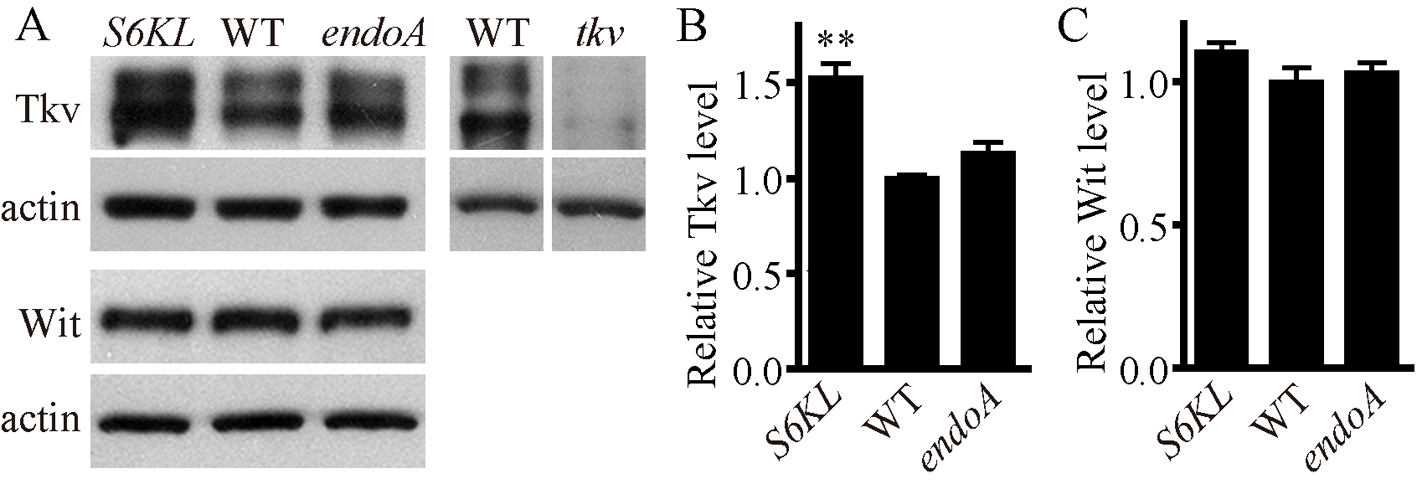

Supplement: S5 Fig — (A) Western results of larval brains from wild type, S6KL 140, and endoA Δ4 /endoA EY02730 mutants probed with anti-Tkv (recognizing multiple Tkv isoforms) and anti-Wit antibodies. The specificity of anti-Tkv was verified in tkv 7/tkv k16713 mutants. Actin was used as a loading control. (B and C) Quantification of the relative protein levels of Tkv (B) and Wit (C) in the larval brains of wild type, S6KL 140, and endoA Δ4 /endoA EY02730 mutants. The level of Tkv was increased in S6KL but not endoA mutants (B); the level of Wit was unaltered in both mutants (C). n = 3, **p<0.01 by Student’s t-tests; error bars indicate SEM. (TIF) [file pgen.1004984.s005.tif]

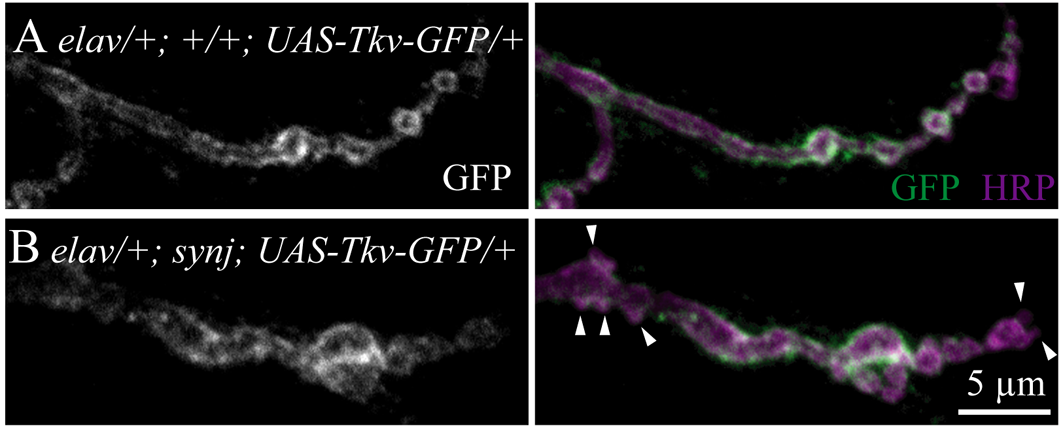

Supplement: S6 Fig — Confocal images of NMJ 4 synapses double-labeled with anti-GFP (green) and anti-HRP (magenta) in control (elav-Gal4/+;+/+;UAS-Tkv-GFP/+) (A) and synaptojanin mutants (elav-Gal4/+;synj 1 /synj Ly ;UAS-Tkv-GFP/+) (B) showing similar Tkv-GFP staining signals. Arrowheads indicate satellite boutons. Scale bar, 5 μm. (TIF) [file pgen.1004984.s006.tif]

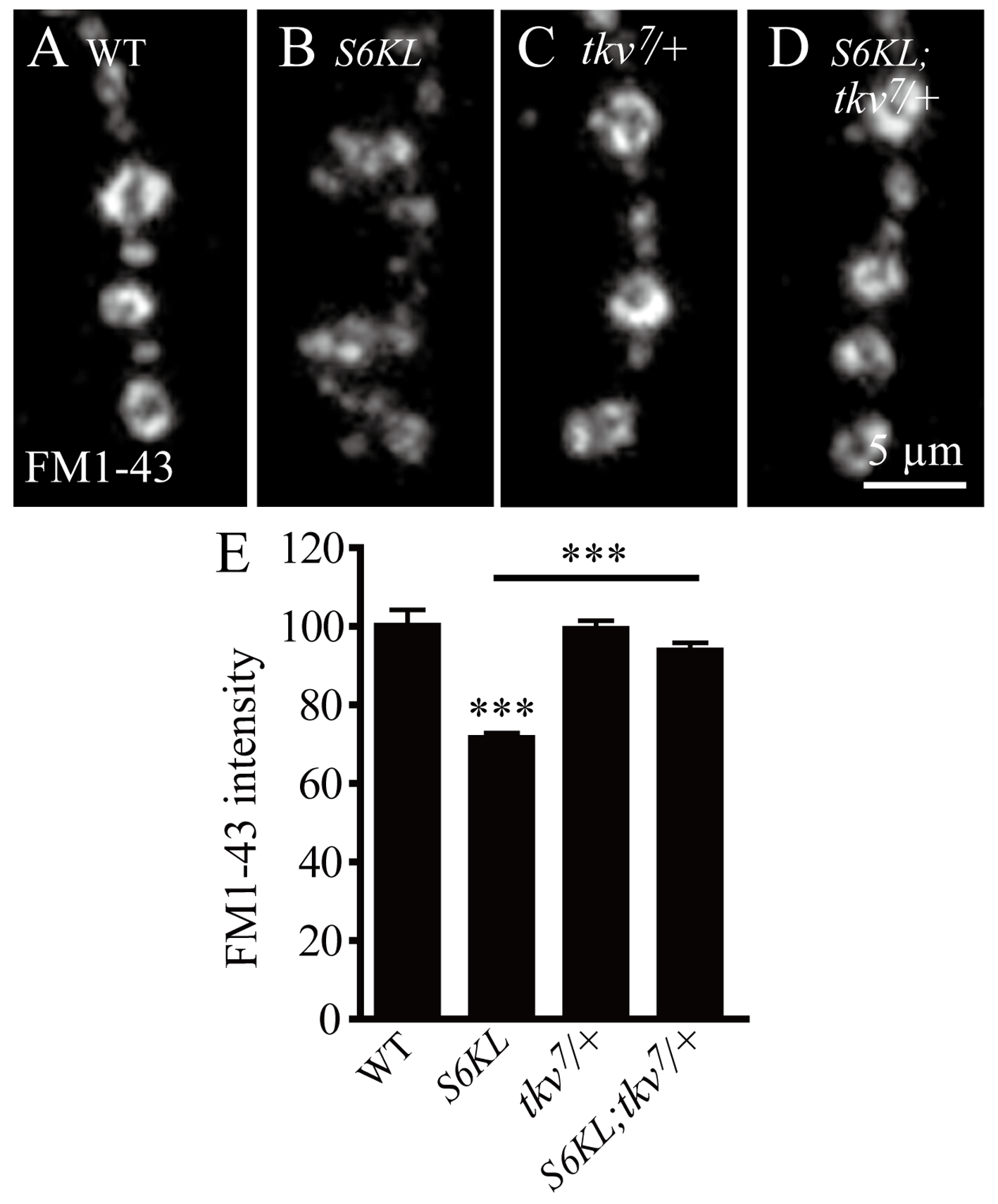

Supplement: S7 Fig — (A–D) NMJ 4 synapses in abdominal segment A3 were loaded with FM1–43 in wild type (A), S6KL 140 (B), tkv 7/+ (C), and S6KL 140 ;tkv 7 /+ (D). Scale bar, 5 μm. (E) Quantification of FM1–43 fluorescence intensities in NMJ boutons following high K+-stimulated endocytosis. n = 29, 20, 26, and 25 NMJs for wild type, S6KL 140, tkv 7/+, and S6KL 140 ;tkv 7 /+, respectively. ***p<0.001 by one-way ANOVA with Tukey post hoc test; error bars indicate SEM. (TIF) [file pgen.1004984.s007.tif]

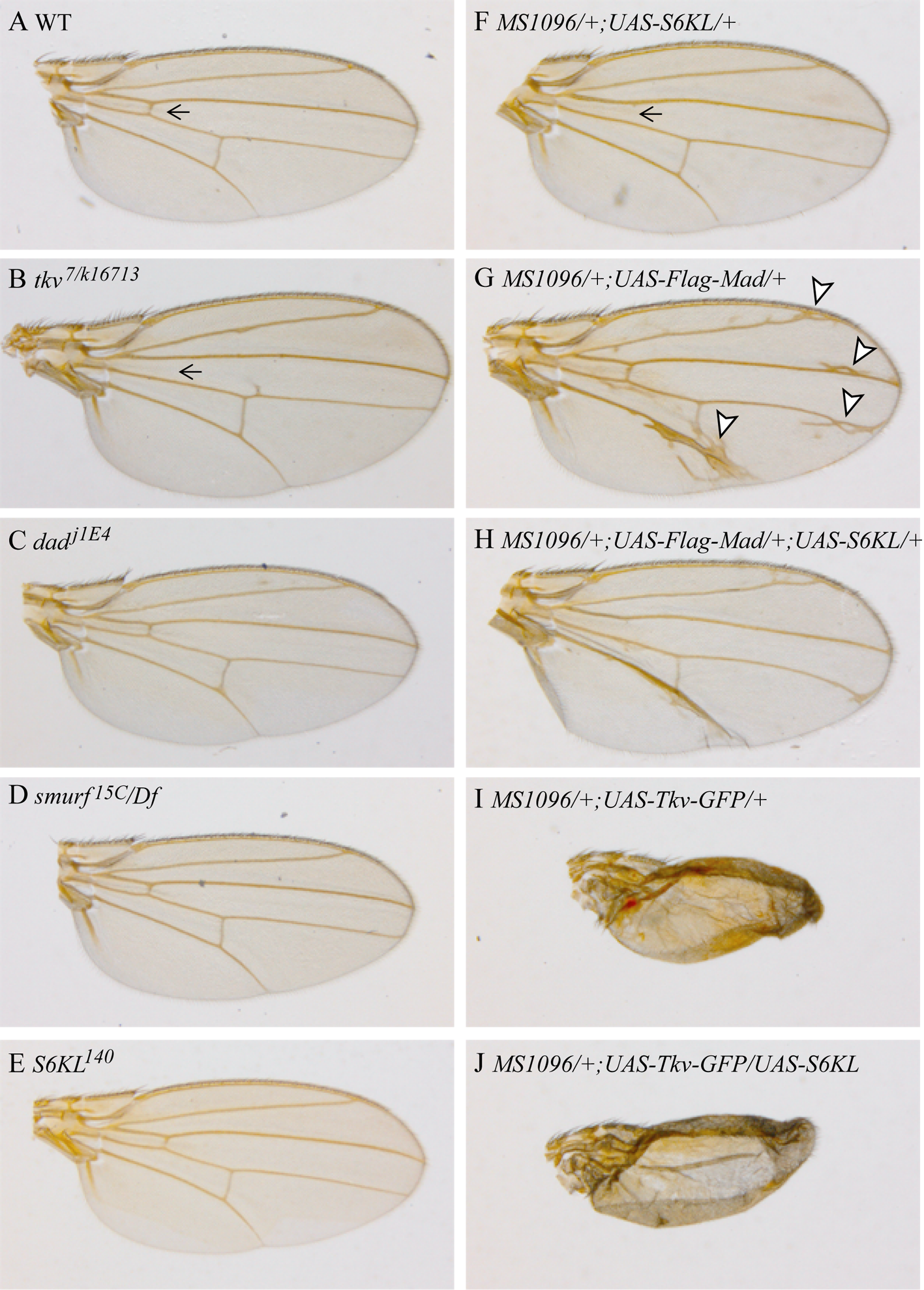

Supplement: S8 Fig — Whole wing images from different genotypes are shown (A-J). dadJ1E4, smurf15C, and S6KL140 mutants showed normal vein pattern and wing morphology. However, overexpression of S6KL throughout wing blade driven by MS1096-Gal4 led to the absence of the anterior cross vein (ACV, indicated by arrows), recapitulating that of tkv mutants (compare B and F). Overexpression of S6KL rescued the ectopic vein phenotypes (indicated by white arrowheads in G) caused by Mad overexpression (compare G and H), but did not rescue the wing phenotype caused by Tkv-GFP overexpression, presumably due to its strong effect (compare I and J). (TIF) [file pgen.1004984.s008.tif]

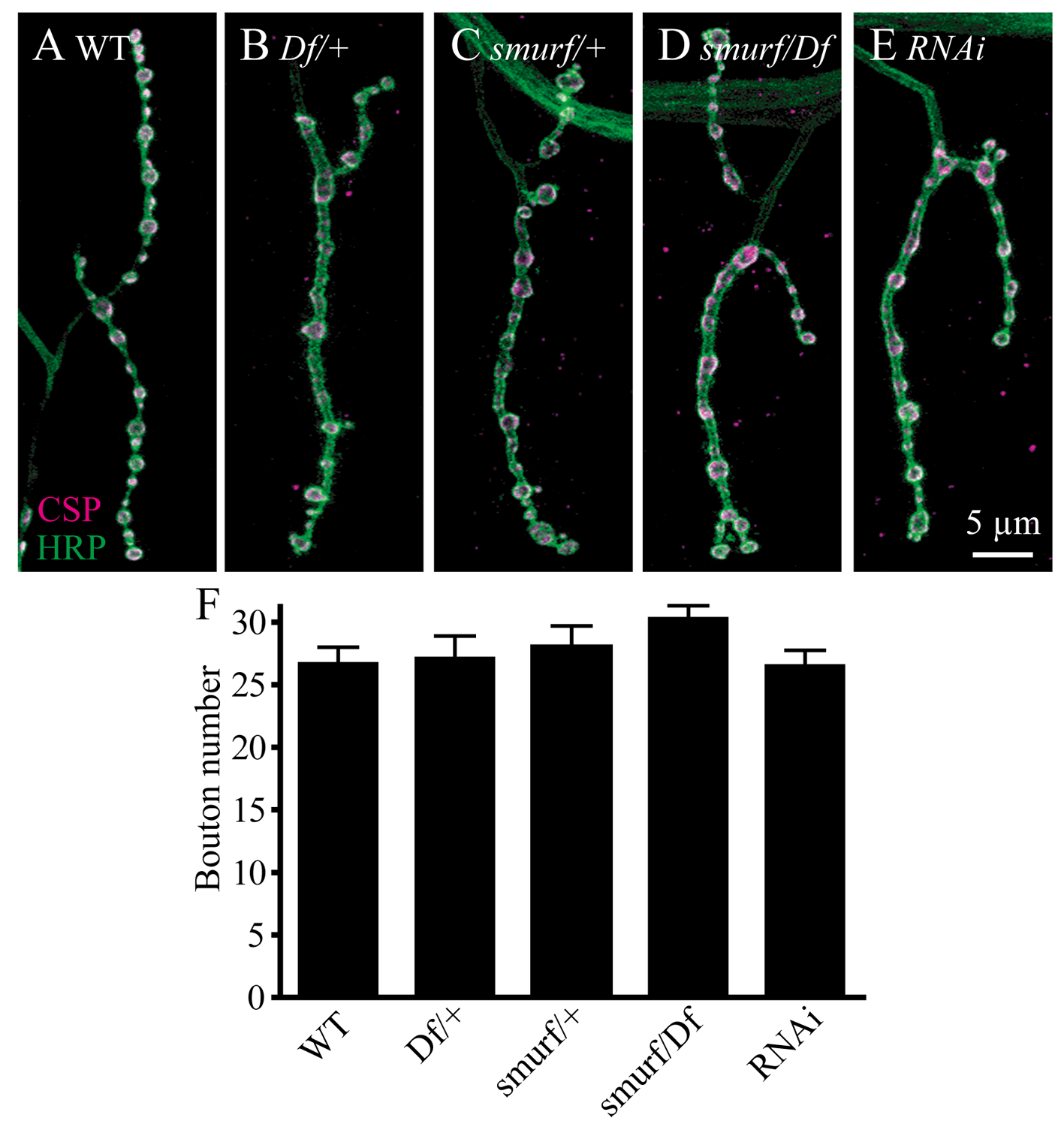

Supplement: S9 Fig — (A–E) Representative NMJ 4 synapses of different genotypes double-stained with anti-HRP recognizing neuronal plasma membrane (green) and an antibody against CSP (magenta), a synaptic vesicle protein. The genotypes are: WT (A), Df(2R)Exel7149/+ (B), smurf 15c /+ (C), hemizygous smurf 15c /Df(2R)Exel7149 (D), and elav/+; Smurf RNAi/+ (E). Scale bar, 5 μm. (F) Statistical results of the number of total boutons in different genotypes. n = 18, 12, 16, 30 and 13 NMJs for WT, Df(2R)Exel7149/+, smurf 15c /+, smurf 15c /Df(2R)Exel7149, and elav/+; Smurf RNAi/+, respectively. Error bars indicate SEM. (TIF) [file pgen.1004984.s009.tif]
