# Supplementary material for: Drosophila S6 Kinase Like Inhibits Neuromuscular Junction Growth by Downregulating the BMP Receptor Thickveins
Source: PLoS Genet. 2015 Mar 6;11(3):e1004984. doi: 10.1371/journal.pgen.1004984 (PMC4351882; doi:10.1371/journal.pgen.1004984)
Supplement: S1 Text — (DOCX) [file pgen.1004984.s010.docx]

**Supplemental methods**

***Drosophila* stocks and antibodies:**

*Drosophila* stocks including *dad-lacZ* [1], *endoA^EY02730^* [2], *endoA^Δ4^*, *synj^1^*, *Df(2R)Exel7149/CyO* (*Df(2R)Exel7149* deletes *smurf* completely), *MS1096-Gal4*, and *Vg-Gal4* were obtained from the Bloomington Stock Center. *Smurf* RNAi line (THU5271) was obtained from Tsinghua University. *synj^Ly^*, *smurf^15c^*, and *UAS-Flag-Mad* have been described previously [3-5].

Anti-TkvC (f3-5) was supplied by Dr. M.B. O’Connor (University of Minnesota, Minneapolis), while anti-Endophilin A and anti-Eps15 were provided by Dr. H. Bellen (Baylor College of Medicine, Houston). Mouse anti-β-Gal and anti-Dynamin were purchased from Promega (1:1000) and BD Transduction Laboratories (1:200), respectively.

**Quantification of *tkv* mRNA level.** Quantification of *tkv* mRNA levels was performed largely according to a published protocol [6]. In brief, total RNA was isolated from third instar larval brains and ventral ganglia of the various genotypes using Rneasy mini kit (QiaGen) according to the manufacturer’s instructions. Total RNA was reverse transcribed into single-stranded cDNA using Super Script III First-Strand Synthesis System (Invitrogen). Quantitative PCR was performed using the Agilent Mx3000p real-time PCR detection system and the Power SYBR® Green PCR Master Mix (Applied Biosystems). The primers for detecting *tkv* mRNA were 5’-AGTGGGTCTCGTTCTGTGGG-3’ and 5’-GTGATAGGGCAGGGCGTAGT-3’. The primers 5’-GCTGAGCGTGAAATCGTCCGTG-3’and 5’-CCCAAGAACGAGGGCTGGAACA-3’ were used to detect actin mRNA. The expression level of *tkv* mRNA was normalized to that of actin mRNA. At least four biological repeats were performed for statistical analysis

**Reference**

1. Tsuneizumi K, Nakayama T, Kamoshida Y, Kornberg TB, Christian JL, et al. (1997) Daughters against dpp modulates dpp organizing activity in Drosophila wing development. Nature 389: 627-631.

2. O'Connor-Giles KM, Ho LL, Ganetzky B (2008) Nervous wreck interacts with thickveins and the endocytic machinery to attenuate retrograde BMP signaling during synaptic growth. Neuron 58: 507-518.

3. Oh H, Irvine KD (2011) Cooperative regulation of growth by Yorkie and Mad through bantam. Dev Cell 20: 109-122.

4. Podos SD, Hanson KK, Wang YC, Ferguson EL (2001) The DSmurf ubiquitin-protein ligase restricts BMP signaling spatially and temporally during Drosophila embryogenesis. Dev Cell 1: 567-578.

5. Dickman DK, Horne JA, Meinertzhagen IA, Schwarz TL (2005) A slowed classical pathway rather than kiss-and-run mediates endocytosis at synapses lacking synaptojanin and endophilin. Cell 123: 521-533.

6. Shi W, Chen Y, Gan G, Wang D, Ren J, et al. (2013) Brain tumor regulates neuromuscular synapse growth and endocytosis in Drosophila by suppressing mad expression. J Neurosci 33: 12352-12363.
